# Identification and Characterization of an *In Silico* Designed Membrane‐Active Peptide with Antiviral Properties

**DOI:** 10.1002/advs.202513911

**Published:** 2026-01-09

**Authors:** Pascal von Maltitz, Niek van Hilten, Tatjana Weil, Thunchanok Thummaraj, Jeroen Methorst, Dennis Aschmann, Alexander Kros, Clarissa Read, Jasmina Gačanin, Herre Jelger Risselada, Jan Münch

**Affiliations:** ^1^ Institute of Molecular Virology Ulm University Medical Center Ulm Germany; ^2^ Department of Pharmaceutical Chemistry Cardiovascular Research Institute University of California San Francisco USA; ^3^ Max Planck Institute for Polymer Research Mainz Germany; ^4^ Department of Physics Technical University Dortmund Dortmund Germany; ^5^ Leiden Institute of Chemistry Leiden University Leiden The Netherlands; ^6^ Central Facility for Electron Microscopy Ulm University Ulm Germany

**Keywords:** AMP, antiviral peptide, broad‐spectrum, membrane‐active

## Abstract

Broad‐spectrum antivirals are urgently needed to counter emerging viral threats. Targeting the viral envelope, an essential, conserved, and host‐derived structure, offers a promising strategy with a low risk of resistance. Here, we report the in silico design and experimental characterization of P1.6, a 24‐mer peptide generated using an evolutionary molecular dynamics (Evo‐MD) platform and optimized to sense and exploit lipid packing defects in viral membranes. Among nine Evo‐MD–derived candidates, P1.6 showed the strongest membrane‐disruptive activity and inhibited HIV‐1, Zika virus, and herpes simplex viruses with IC_50_ values ranging from ∼0.06 to 3.5 µm. P1.6 efficiently disrupted virus‐like liposomes without causing cytotoxicity or hemolysis at antiviral concentrations. All‐atom MD simulations predicted a predominantly α‐helical solution structure with a central kink and flexible termini. Upon membrane engagement, this kink was largely lost, yielding a more continuous and stabilized helix. ATR‐FTIR spectroscopy confirmed the membrane‐induced increase in helicity. Coarse‐grained MD simulations further demonstrated that P1.6 stabilizes transient membrane pores, while electron microscopy of treated HIV‐1 particles revealed extensive envelope rupture and capsid release. Together, these results establish P1.6 as a potent membrane‐active antiviral lead and highlight the utility of Evo‐MD–guided peptide design to target conserved biophysical vulnerabilities in viral envelopes.

## Graphical abstract

An evolutionary molecular dynamics platform is used to design P1.6, a membrane‐active peptide that senses lipid packing defects in viral envelopes. P1.6 adopts a stabilized α‐helical structure upon membrane contact, disrupts virus‐like liposomes, and damages HIV‐1 particles. This study highlights Evo‐MD–guided peptide engineering as a strategy for broad antiviral development.

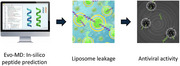

## 1 Introduction

Viruses continue to represent a major and growing threat to global health, as evidenced by recent outbreaks of respiratory viruses including influenza, respiratory syncytial virus (RSV), coronaviruses (such as SARS‐CoV‐2), and arboviruses from the Flaviviridae family. Many of these viruses originate from zoonotic spillover events, facilitated by ecological disruptions and environmental changes that enhance the risk of cross‐species transmission [1]. Climate change further exacerbates this threat by expanding the geographical range of arthropod vectors that transmit pathogenic viruses such as Zika and dengue virus [2]. In addition, the HIV pandemic remains a stark reminder of the long‐term public health and socioeconomic impacts of zoonotic viral infections. Together, these challenges highlight an urgent need for effective antiviral therapeutics capable of providing broad‐spectrum protection against both current and emerging viral threats.

Most existing antiviral therapies are designed to target virus‐specific proteins such as polymerases or viral proteases. While these drugs can be highly effective, their specificity limits their use to a narrow range of viruses. Moreover, their effectiveness is often undermined by the rapid evolution of viral genomes, which can lead to the emergence of drug‐resistant strains. This underscores the need for broad‐spectrum antiviral agents that target conserved viral structures and mechanisms of infection [3].

One promising, yet underexplored, approach is to target the viral envelope, which is essential for the infectivity of many pathogenic viruses. The viral envelope consists of a lipid bilayer derived from host cellular membranes during the budding process. Enveloped viruses acquire their membranes from distinct cellular compartments, including the plasma membrane, the endoplasmic reticulum (ER), the Golgi apparatus, or the ER‐Golgi intermediate compartment (ERGIC) [4]. This lipid bilayer not only provides structural integrity but also embeds viral glycoproteins critical for host cell attachment and membrane fusion. Disruption of the viral envelope leads to irreversible loss of infectivity, as demonstrated by the efficacy of alcohol‐based disinfectants and detergents [5]. However, selectively targeting viral membranes without harming host cells poses significant challenges due to their shared lipid composition. Despite these challenges, key structural differences between viral and host membranes offer opportunities for selective targeting. Viral particles are considerably smaller than cells, resulting in high membrane curvature and tension that increase their susceptibility to physical and chemical disruption [3]. Furthermore, viral budding occurs preferentially at lipid rafts, cholesterol‐ and sphingolipid‐enriched microdomains, which contribute to compositional differences between viral and cellular membranes. Unlike eukaryotic cells, viruses are incapable of repairing membrane damage, making them particularly vulnerable to membrane‐disrupting agents.

These features have been exploited by various classes of molecules, including amphipathic peptides and synthetic compounds, which can interact with and disrupt viral membranes. Antimicrobial peptides (AMPs), for example, are a diverse group of naturally occurring molecules that contribute to innate immunity by destabilizing microbial membranes [6]. Among AMPs, antiviral peptides (AVPs) such as melittin from bee venom, the M2 peptide from influenza virus, and C5A from hepatitis C virus have demonstrated broad‐spectrum activity against enveloped viruses by disrupting membrane integrity [7, 8, 9, 10]. The AH‐peptide, derived from the NS5A membrane anchor domain and structurally related to C5A, has shown protective efficacy against Zika virus infection in murine models [7, 8]. Synthetic molecules such as molecular tweezers have also shown promise as broad‐spectrum antivirals [11, 12, 13]. These supramolecular structures selectively bind to exposed phospholipid head groups in viral membranes, destabilizing the lipid bilayer and leading to viral inactivation. Notably, molecular tweezers exhibit minimal cytotoxicity due to their selective targeting of viral membranes, further validating the concept of membrane‐targeting antivirals as a feasible therapeutic strategy [3].

Despite these advances, rational design and optimization of membrane‐active peptides and compounds remain challenging. The complexity of peptide‐membrane interactions, including steric effects between lipids, membrane tension, and lipid packing defects, makes it difficult to predict peptide behavior and activity based on sequence alone. Traditional experimental screening methods are time‐consuming and often inadequate for addressing rapidly emerging viral threats [10, 14]. To overcome these limitations, computational approaches such as molecular dynamics (MD) simulations have become indispensable tools in the rational design of membrane‐active peptides. MD simulations provide detailed insights into peptide‐membrane interactions at atomic and coarse‐grained levels, revealing mechanisms such as pore formation and membrane destabilization [15, 16, 17]. Recent advances have further combined MD simulations with evolutionary algorithms, enabling the iterative optimization of peptide sequences for enhanced membrane affinity and specificity. Evo‐MD is one such approach that integrates directed evolution with MD simulations to design and refine amphipathic peptides capable of sensing and exploiting lipid packing defects associated with highly curved viral membranes [18, 19, 20].

In this study, we applied the Evo‐MD approach to design *de novo* amphipathic peptides with selective activity against lipid bilayers. Through a combination of molecular dynamics simulations, coarse‐grained modeling, and genetic algorithms, we identified and optimized peptide sequences predicted to interact with and destabilize lipid bilayers mimicking viral envelopes. We synthesized and characterized a lead peptide, P1.6, which demonstrated potent antiviral activity against multiple enveloped viruses, including HIV‐1, Zika virus (ZIKV), and herpes simplex virus (HSV), while exhibiting minimal cytotoxicity. Our findings highlight the potential of computationally guided strategies to accelerate the discovery of broad‐spectrum antiviral agents that target conserved structural vulnerabilities in viral membranes.

## 2 Results

### 2.1 Identification of a Membrane‐Active Peptide by Evo‐MD

We designed a library of *de novo* antiviral peptides targeting viral envelopes by evolving their lipid defect‐sensing properties through an iterative molecular dynamic‐based algorithm (Evo‐MD, Figure S1). In this approach, peptide sequences were subjected to cycles of random mutation and selection in coarse‐grained MD simulations, favoring candidates that preferentially insert into lipid packing defects [19]. Since membrane‐surface peptides are predominantly α‐helical and the coarse‐grained MD setup does not model protein folding events, we assumed a fixed α‐helical secondary structure when generating the starting conformations. To further reduce the conformational search space, ten amino acids with low α‐helical propensities were excluded from the design process [18, 21]. Using this strategy, peptides were constructed from repeating 24‐amino‐acid motifs and refined over more than 50 in silico generations. From the Evo‐MD predictions, we selected nine 24‐mer peptide variants (P1.1–P1.9; see Table S1) for experimental characterization in biophysical and antiviral assays.

**FIGURE 1:**
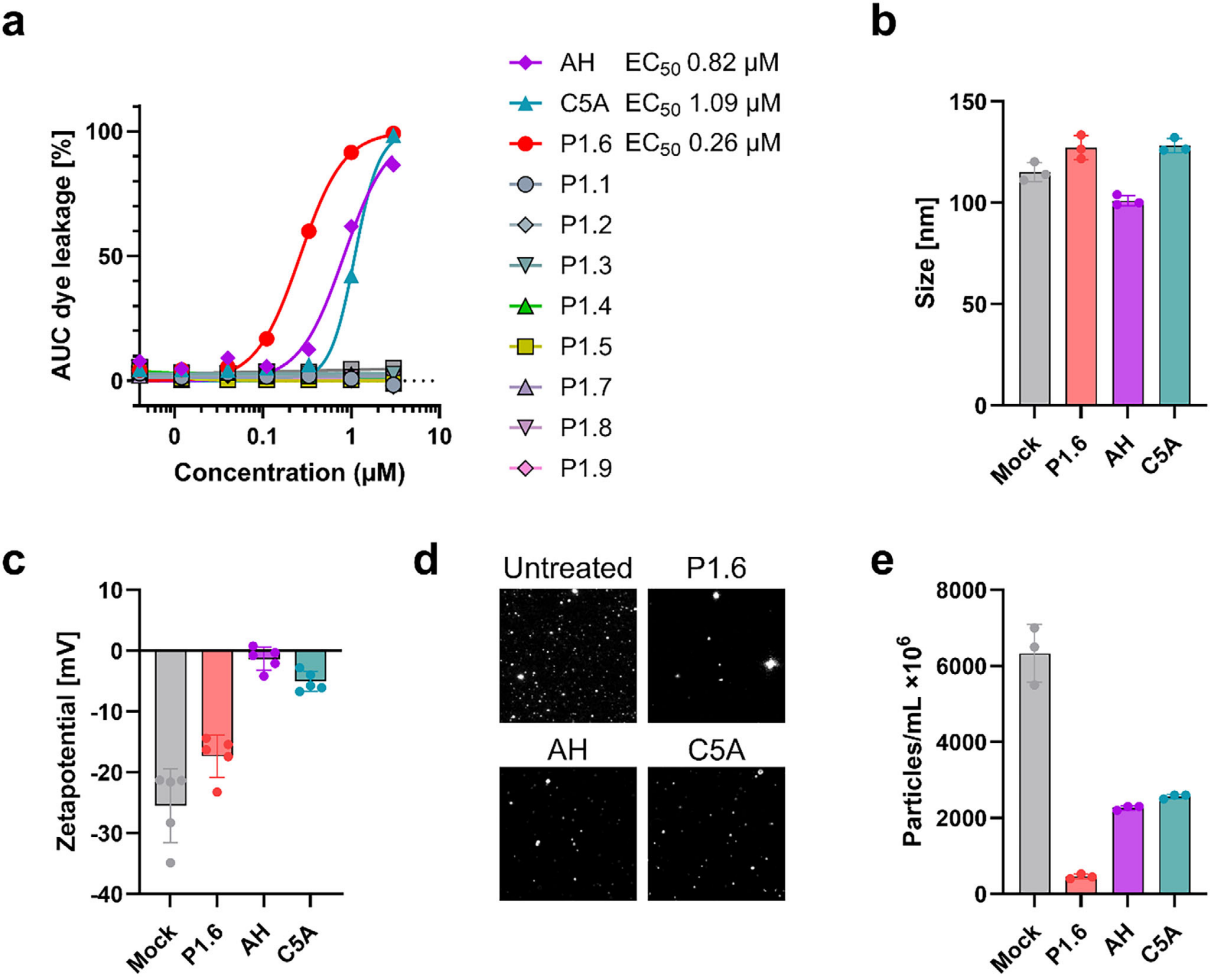
Liposome disruption and biophysical characterization after peptide treatment. (a) Area under the curve (AUC) of carboxyfluorescein dye release from liposomes upon exposure to peptides P1.1–P1.9, AH, C5A, or solvent control (DMF) for 30 min. Fluorescence was recorded over time, and AUC values were normalized to 100% lysis induced by Triton X‐100. EC_50_ values for P1.6, AH, and C5A are indicated. Shown are the results of one experiment performed in triplicate ± SD. (b, c) Mean hydrodynamic size and zeta potential of DOPC (45 mol%), sphingomyelin (25 mol%), and cholesterol (30 mol%) liposomes before and after peptide treatment (3 µm), determined by nanoparticle tracking analysis (NTA). Shown are the results of one experiment measured at 11 positions per cycle in three replicate runs ± SD. (d) Representative NTA snapshots illustrating the reduction in particle counts after peptide exposure. (e) Particle number of liposomes before and after treatment with P1.6, AH, or C5A (3 µm), measured by NTA.

To identify the most active membrane‐disruptive peptide from the Evo‐MD library, we first screened all nine designed candidates (P1.1–P1.9) using a carboxyfluorescein leakage assay (Figure 1a) [11]. Among these peptides, P1.6 induced the strongest and fastest dye release, with an EC_50_ of ∼0.26 µm. Other variants showed substantially lower activity, and none approached the potency of P1.6. For comparison, the benchmark peptides AH and C5A displayed EC_50_ values of ∼0.82 and ∼1.09 µm, respectively, confirming P1.6 as the most active peptide in the screen (Figure 1a). Based on this clear superiority, P1.6 was selected for further biophysical characterization.

To characterize how the peptides affect membrane integrity, we first analyzed virus‐like liposomes by nanoparticle tracking analysis (NTA). Untreated DOPC/SM/Chol vesicles displayed a uniform hydrodynamic diameter of ∼115 nm (Figure 1b). Incubation with P1.6, AH, or C5A did not alter the mean particle size, indicating that the peptides do not cause vesicle swelling, fusion, or aggregation. Instead, their effects are expected to manifest as changes in membrane stability or vesicle integrity.

We next assessed peptide/membrane interactions by measuring the zeta potential of the liposomes (Figure 1c). As expected for vesicles containing phosphatidylcholine (PC), the untreated liposomes exhibited a negative zeta potential. This well‐established property results from the asymmetric exposure of the PC headgroup: the phosphate moiety is more solvent‐exposed than the choline group, generating a net negative electrostatic potential at the shear plane [22, 23]. The cholesterol present in our DOPC/SM/Chol formulation further enhances this effect by increasing lipid packing order and orienting headgroups toward the aqueous phase [23]. Relative to this baseline, all three peptides reduced the magnitude of the negative zeta potential, consistent with surface binding and partial shielding of exposed phosphate groups. Differences in the degree of the zeta‐potential shift likely reflect peptide‐specific features such as charge distribution, size, and depth of membrane engagement.

To directly assess vesicle integrity, we examined particle numbers by NTA. Particle quantification together with representative NTA snapshots (Figure 1d) revealed a pronounced loss of detectable liposomes following peptide treatment. P1.6 caused the strongest reduction in vesicle number (Figure 1e), consistent with efficient membrane rupture. AH and C5A also decreased particle counts, but to a lesser extent. Importantly, these reductions in particle abundance occurred without changes in particle size, indicating that vesicles were physically destroyed rather than fused or deformed.

Together, these data show that P1.6 binds to the membrane surface, perturbs lipid headgroup organization, and efficiently disrupts liposome integrity.

### 2.2 Predicted Structure of P1.6

**FIGURE 2:**
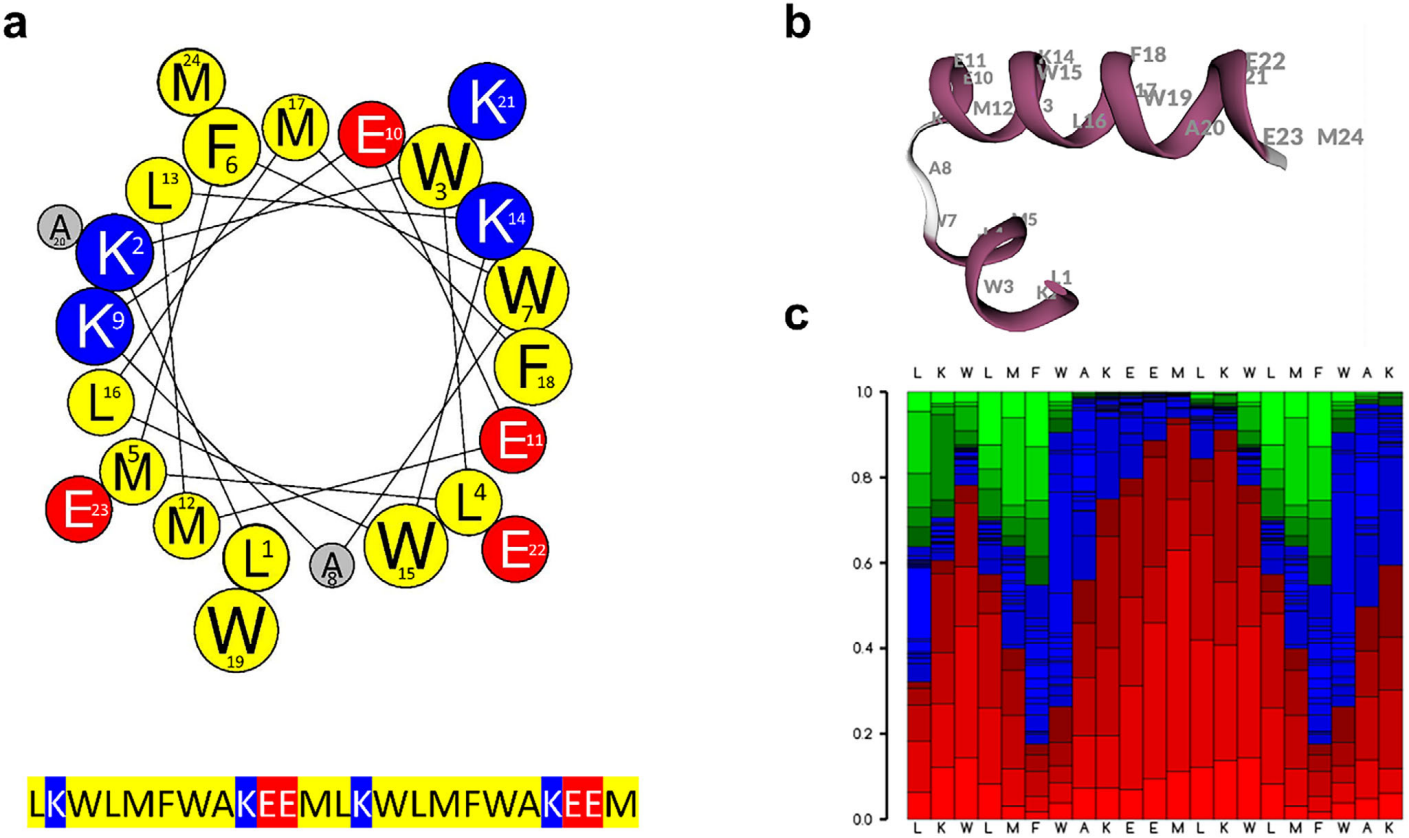
Structural characterization of P1.6. (a) Helical wheel projection of P1.6 generated using HeliQuest, illustrating the α‐helical structure. Hydrophobic residues are shown in yellow, positively charged residues in blue, negatively charged residues in red, and others in grey. The sequence is displayed below with color‐coded residue types. (b) Ribbon diagram of the lowest‐energy conformer of P1.6, revealing a kinked α‐helical structure. (c) Heatmap of secondary structure probabilities for each residue in the P1.6 sequence, indicating the likelihood of α‐helical (red), extended (blue), and random coil (green) conformations (PEP‐FOLD3 server).

To gain insight into the structural basis of P1.6's membrane activity, we performed in silico modeling of its primary and secondary structure. A helical wheel projection (HeliQuest) revealed a predominantly hydrophobic α‐helical profile, characterized by a distinct segregation of charged residues (primarily lysine and glutamic acid) along the helix (Figure 2a). Further secondary structure predictions using the PEP‐FOLD3 server indicated that P1.6 adopts a kinked α‐helical conformation in solution (Figure 2b). Notably, helix‐kink‐helix structures have been shown to enhance the antimicrobial activity of α‐helical peptides when compared to their unkinked counterparts [24]. This enhanced activity might be linked to the ability of kinked helices to influence membrane pore formation, specifically by stabilizing toroidal pores, which require the structural flexibility conferred by the kinked architecture [24, 25]. The model also displays a central hydrophobic core enriched in bulky aromatics and leucine/methionine residues, features that likely facilitate deep membrane insertion and local destabilization of the bilayer [26]. To evaluate the stability of this conformation across the sequence, we generated a heatmap of secondary structure propensities for each residue of P1.6 (Figure 2c). This analysis confirmed a high likelihood of α‐helical structure throughout most of the peptide, especially in the central and C‐terminal region, whereas the N‐terminus displayed greater flexibility with a tendency toward random coil or extended conformations. Together, these in silico analyses support the hypothesis that the potent membrane‐disrupting activity of P1.6 relies on an α‐helix optimized for membrane interaction.

### 2.3 Experimental Validation of P1.6 Secondary Structure by ATR‐FTIR

**FIGURE 3:**
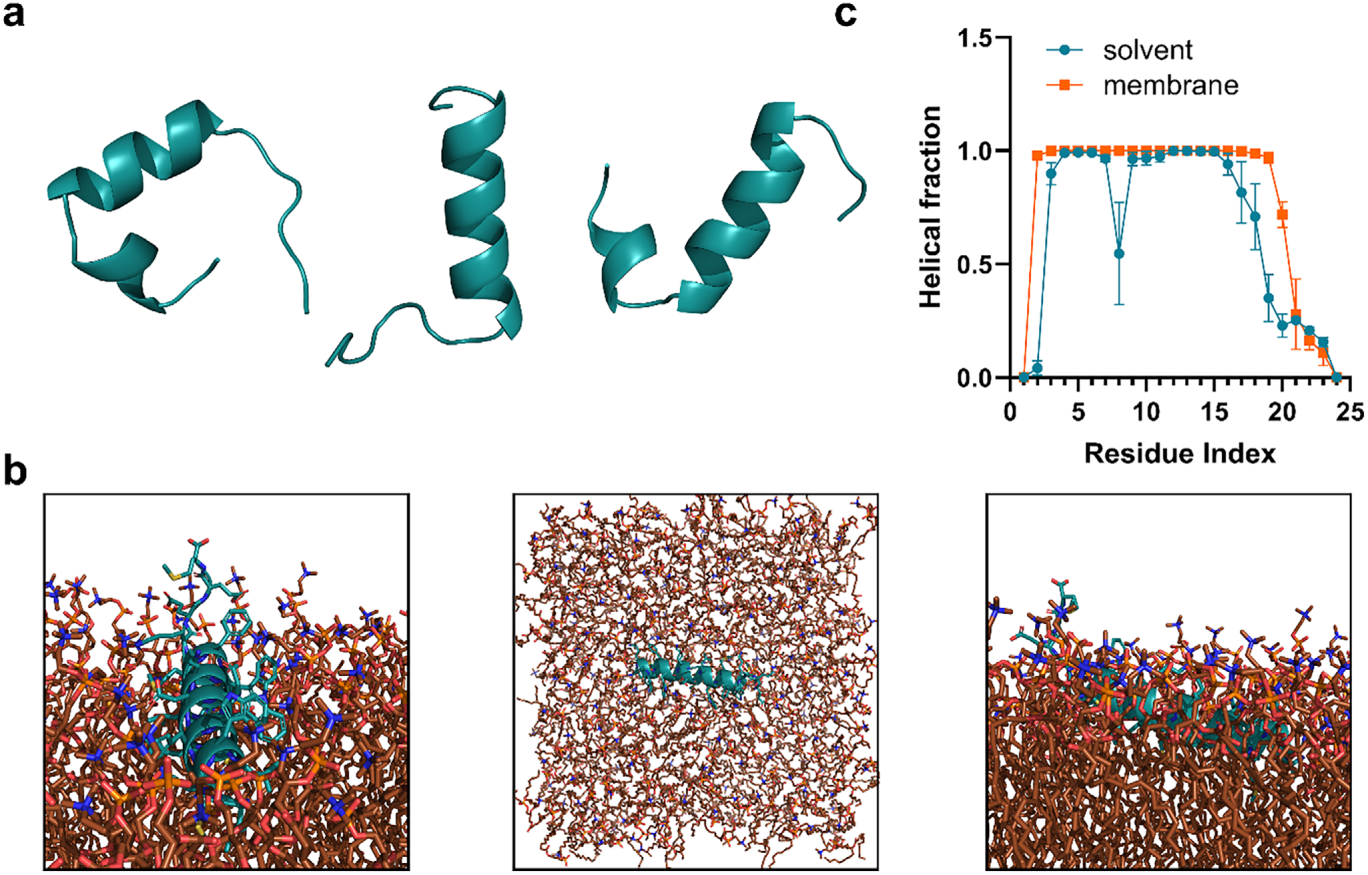
Structural dynamics and residue‐wise α‐helicity of peptide P1.6 in aqueous and membrane environments. (a) Representative conformations of P1.6 (cyan) in water showing partial disorder at the termini and, in two of the replicates, a pronounced kink around residues 7–11. (b) Representative atomistic snapshots of P1.6 (cyan) bound to a POPC bilayer (orange) supplemented with cholesterol (blue). The helical segment inserts into the membrane core, while the unstructured region remains solvent‐exposed. Water and ions are omitted for clarity. From left to right, the images show front, top, and side views of the peptide within the membrane. (c) Per‐residue α‐helicity of P1.6 in solution and in contact with a POPC membrane, determined by the DSSP algorithm. The graphs represent the mean helix fraction in three replicates ± SEM.

Given that many antimicrobial peptides, including magainin and melittin, commonly adopt α‐helical structures upon membrane contact [27, 28], we first performed all‐atom molecular dynamics simulations of P1.6 in aqueous solution, followed by simulations in the presence of a pure POPC membrane. In water, P1.6 adopted a partially disordered structure, with flexible termini and a pronounced kink in two of the three replicates (Figure 3a). Upon interaction with the POPC membrane, the peptide engaged with the lipid headgroups and underwent partial insertion. The helical region is embedded into the membrane core, while the disordered segment remained solvent‐exposed (Figure 3b).

The per‐residue α‐helicity profile (Figure 3c), calculated using the DSSP algorithm, revealed a discontinuous helical structure with reduced helicity localized between residues 7–11 (with respect to time, see Figure S10b) with an overall helicity of 69.5 % ± 1.8%, based on the area under the curve (AUC). The corresponding helicity analysis in the membrane‐bound state indicated a more continuous and stabilized α‐helix across all central residues, supporting a membrane‐induced structural ordering of P1.6 (with respect to time, see Figure S10a) with an overall helicity of 83.5% ± 1%. Additionally, the change in folding between the membrane‐bound and soluble states is reflected by significant changes in intramolecular interactions, as evidenced by the measurement of intrapeptide hydrogen bond occupancy (Figure S8).

**FIGURE 4:**
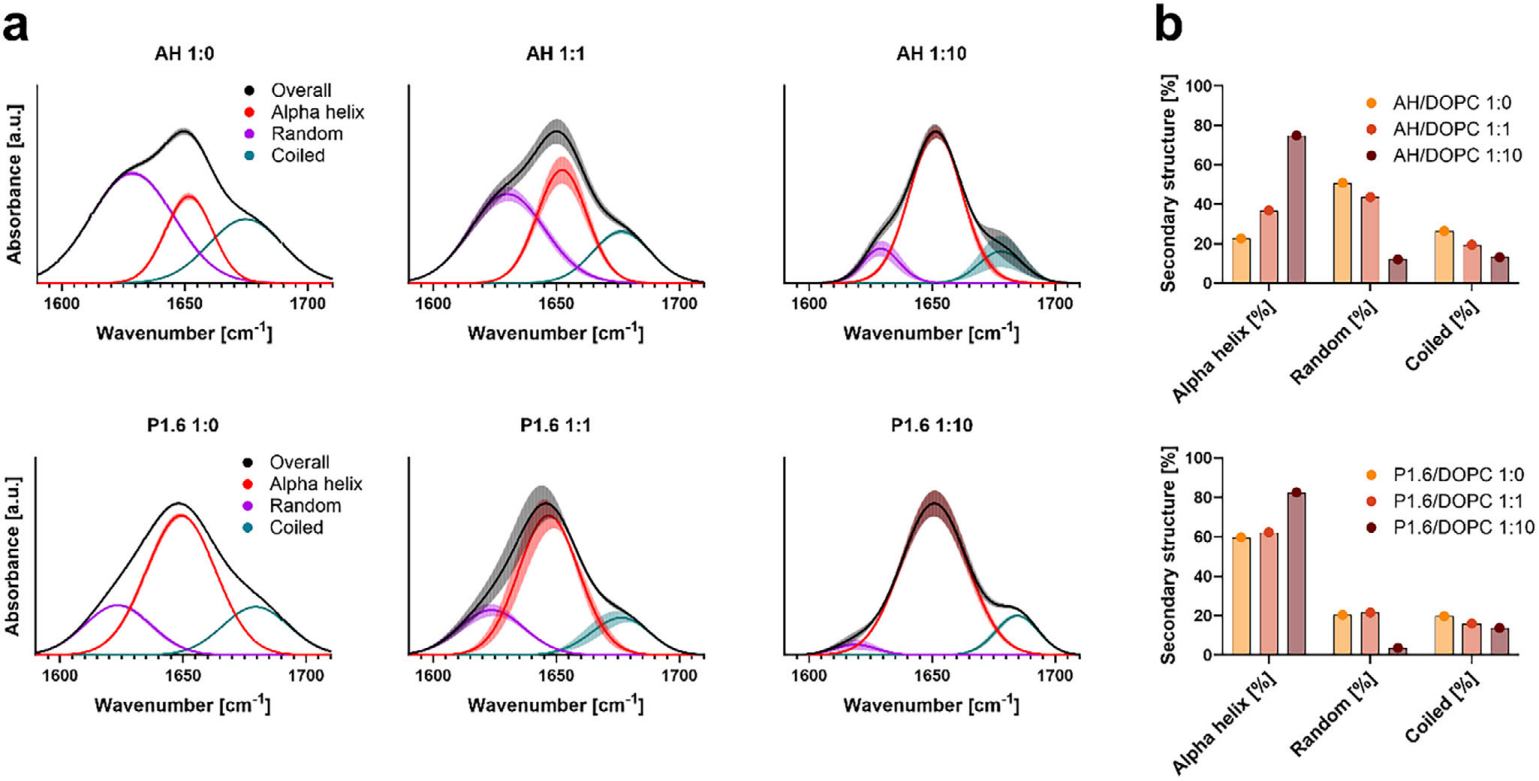
FTIR analysis of P1.6 secondary structure in the presence of DOPC liposomes. (a) FTIR spectra and peak deconvolution of AH (top panels) and P1.6 (bottom panels) at peptide‐to‐lipid molar ratios of 1:0 (no lipid), 1:1, and 1:10, for 0:10 (no peptide), see Figure S6. Colored curves represent the overall spectrum (black), α‐helix (red), random coil (magenta), and β‐sheet/coiled structures (cyan). (b) Quantitative analysis of secondary structure components, shown as percentage area under the curve for AH (top graph) and P1.6 (bottom graph), derived from spectral deconvolution in (a). Data represent mean ± SEM from one experiment performed in triplicate. The y‐axis shows arbitrary units corresponding to spectral intensity, normalized to the mean peak intensity of the overall spectra.

To experimentally validate the membrane‐induced structural transition, we employed attenuated total reflectance Fourier‐transform infrared (ATR‐FTIR) spectroscopy to assess the secondary structure of P1.6 in the presence of lipid membranes. P1.6 was examined in the absence and presence of lipid (DOPC liposomes) at peptide‐to‐lipid (P:L) ratios of 1:0 (aqueous solution), 1:1, and 1:10. Its FTIR spectra were compared to those of the HCV‐derived AH peptide within the same experimental setup under reported conditions. In solution (P:L 1:0), P1.6 showed a dominant amide I band near 1650 cm^−1^, corresponding to ∼60% α‐helical content (Figure 4a, lower panel), a high helicity even without lipid that corroborates the predicted pre‐formed helix. Upon lipid addition, P1.6 helical content further increased, reaching roughly 80% at P:L 1:10 (Figure 4b, lower graph). This finding indicates that membrane association stabilizes and enhances the helical structure of P1.6. In contrast, the control peptide AH exhibited a classical membrane‐induced folding behavior. AH was mostly unstructured in solution (∼20% helix) but underwent a marked conformational transition upon lipid binding, achieving ∼70% α‐helix at P:L 1:10 (Figure 4a, upper; Figure 4b, upper). This lipid‐triggered folding suggests that AH requires membrane contact to adopt its bioactive helical form, unlike P1.6, which is largely pre‐folded. Spectral deconvolution confirmed that increasing lipid shifted the AH amide I signal from random coil (∼1635 cm^−1^) and other structures, as well as coiled (1660–1700 cm^−1^) toward 1650 cm^−1^ (α‐helix). It should be noted that the peak deconvolution reflects an approximate, visually guided interpretation of the secondary structure distribution; while the results align with trends observed in the raw spectra, they are not fully stable or quantitatively robust. Rather, the deconvolution of normalized spectra was performed to provide a rough estimate of the relative structural contributions. Collectively, the FTIR results verify that P1.6 behaves as a typical α‐helical antiviral peptide: it already maintains a helical secondary structure in aqueous solution that becomes further increased upon membrane interaction.

### 2.4 Cytotoxicity and Hemolytic Activity of P1.6

Before evaluating antiviral efficacy, we assessed the cytotoxicity of P1.6 to ensure active concentrations would not be cell‐lethal. P1.6 exhibited no cytotoxicity across the majority of mammalian cell lines relevant to our infection models (TZM‐bl, Caco‐2, Huh‐7, ELVIS, and Vero E6). All tested cell lines maintained over 80% viability at P1.6 concentrations up to 20 µm (Figure S2). A slight cytotoxic effect was observed only in ELVIS cells at the highest concentration tested. This data indicates that P1.6 is broadly well‐tolerated under the tested conditions. By comparison, the peptide C5A caused substantial cytotoxicity in some cells (reducing TZM‐bl and Vero E6 viability by up to ∼50% at 20 µm), whereas AH showed no significant cytotoxic effect at the same concentration (Figure S2).

**FIGURE 5:**
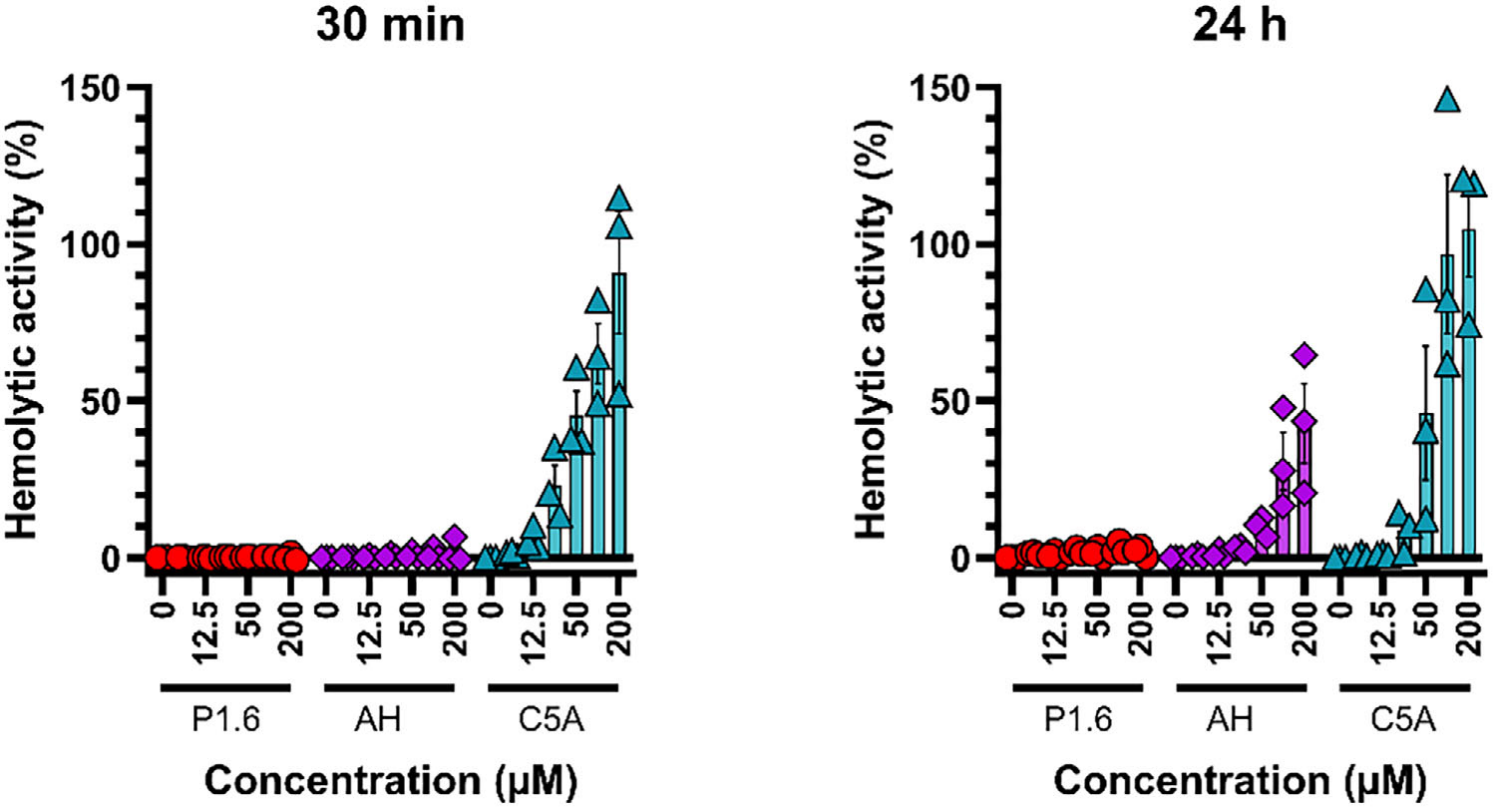
Hemolytic activity of P1.6, AH, and C5A. Human erythrocytes were incubated with increasing concentrations of peptides for 30 min (left) or 24 h (right). Hemolysis was quantified by measuring hemoglobin release via spectrophotometry, normalized to total release induced by Triton X‐100 (1%). Data represent mean ± SEM from three donors, each tested in triplicate.

We next examined the hemolytic activity of P1.6 using human erythrocytes as a model for membrane disruption of host cells. No hemolysis was detected with P1.6 after 30 min or 24 h of incubation, even at the highest tested concentration (Figure 5a,b). Similarly, AH was non‐hemolytic at 30 min but induced about 50% hemolysis after 24 h at the highest concentration tested. In stark contrast, C5A caused strong, dose‐dependent hemolysis within 30 min of exposure (Figure 4). The pronounced hemolytic activity of C5A (and lack thereof for P1.6) underscores the improved safety profile of P1.6. These results indicate that P1.6 can be used at therapeutically relevant concentrations without significant toxicity to human cells or erythrocytes.

### 2.5 Antiviral Activity of P1.6

**FIGURE 6:**
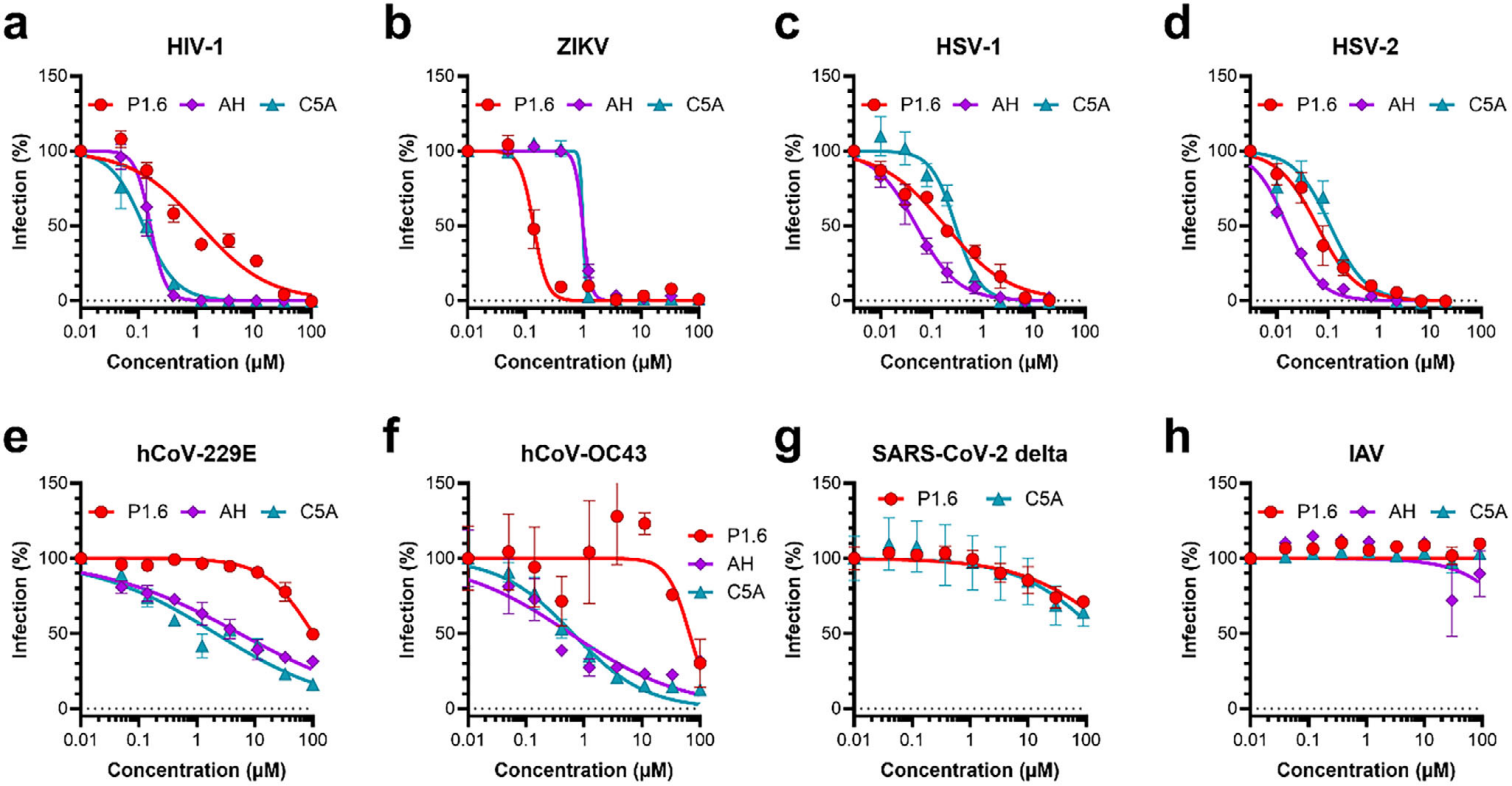
Antiviral activity of P1.6 against enveloped viruses. (a) HIV‐1, (b) ZIKV, (c) HSV‐1, (d) HSV‐2, (e) hCoV‐OC43, (f) hCoV‐229E, (g) SARS‐CoV‐2 Delta, and (h) Influenza A virus (IAV) were pre‐incubated with the indicated concentrations of P1.6, AH, or C5A prior to infection of the respective target cell lines. Infection levels were quantified using reporter gene assays or in‐cell ELISA, as detailed in the Materials and Methods section. Data for HIV‐1, ZIKV, and HSV‐1/2 represent mean ± SEM from three independent experiments; data for respiratory viruses (OC43, 229E, SARS‐CoV‐2, and IAV) are from one representative experiment performed in triplicate ± SD.

Following the favorable safety profile of P1.6, we evaluated its antiviral efficacy against a panel of enveloped viruses, in direct comparison to AH and C5A. The test panel included HIV‐1, Zika virus (ZIKV), two herpesviruses (HSV‐1 and HSV‐2), two human coronaviruses (hCoV‐229E and hCoV‐OC43), the SARS‐CoV‐2 (Delta variant), and influenza A virus (IAV). Antiviral assays were performed in the appropriate cell infection models, and IC_50_ values were determined from dose‐response curves (Figure 6; Table 1).

**TABLE 1:** Antiviral potency (IC_50_ values) of P1.6, AH, and C5A against diverse enveloped viruses.

	IC_50_ [µm]		
	P1.6	AH	C5A
ZIKV	0.28	1.19	0.79
HIV‐1	2.54	0.10	0.13
HSV‐1	0.18	0.05	0.29
HSV‐2	0.06	0.02	0.11
hCoV‐229E	>100	5.48	2.13
hCoV‐OC43	65.86	0.57	0.64
SARS‐CoV‐2	>100	n.a.	>100
IAV	>100	>100	>100

IC_50_ Values Were Calculated From Data Shown in Figure 6; “>100” Indicates no Significant Inhibition at Concentrations of 100 µm.

P1.6 exhibited a dose‐dependent inhibitory effect on HIV‐1 infection in TZM‐bl cells, with an IC_50_ of 2.54 µm (Figure 6a). In comparison, both control peptides, AH and C5A, were more potent, inhibiting HIV‐1 replication with IC_50_ values of 0.10 and 0.13 µm, respectively (Figure 6a). Against ZIKV, P1.6 demonstrated superior antiviral activity relative to both control peptides. In Vero E6 cells, P1.6 inhibited ZIKV infection with an IC_50_ of 0.28 µm, whereas AH and C5A exhibited lower potency, with IC_50_ values of 1.19 and 0.79 µm, respectively (Figure 6b; Table 1). These findings highlight P1.6 as the most effective peptide in this model for targeting ZIKV. P1.6 also effectively inhibited herpes simplex virus infections. For HSV‐1, P1.6 displayed an IC_50_ of 0.18 µm in ELVIS cells (Figure 6c, Table 1). Although AH was more potent with an IC_50_ of 0.05 µm, C5A was less effective (IC_50_ = 0.29 µm). A similar trend was observed for HSV‐2, where P1.6 showed high potency with an IC_50_ of 0.06 µm (Figure 6d, Table 1). Again, AH was slightly more effective (IC_50_ = 0.02 µm), while C5A had an IC_50_ of 0.11 µm (Figure 6d, Table 1).

In contrast, P1.6 showed limited activity against respiratory coronaviruses. For hCoV‐229E, no significant inhibition was observed at concentrations up to 100 µm, while AH and C5A exhibited moderate antiviral activity with IC_50_ values of 5.48 and 2.13 µm, respectively (Figure 6e, Table 1). A similar pattern was noted for hCoV‐OC43, where P1.6 displayed weak activity with an IC_50_ of 65.86 µm, whereas AH and C5A were considerably more effective (IC_50_ = 0.57 and 0.64 µm, respectively) (Figure 6f, Table 1). No significant antiviral activity was observed for P1.6 against the SARS‐CoV‐2 Delta variant and IAV. Neither P1.6 nor C5A inhibited SARS‐CoV‐2 replication at the tested concentrations (Figure 6g). Similarly, all three peptides were inactive against IAV, with IC_50_ values exceeding 100 µm (Figure 6h).

### 2.6 P1.6 Disrupts Membranes Across a Wide Size Range with Enhanced Efficiency on Highly Curved Vesicles

The variability in P1.6 efficacy across viruses prompted us to investigate whether membrane curvature and particle size influence its membrane‐disruptive potential. In particular, smaller virions exhibit higher curvature and more lipid packing defects, which could facilitate peptide action. To test this, we prepared model liposomes spanning four size classes: small unilamellar vesicles of approximately 50, 100, and 200 nm diameter (made by extrusion), and giant unilamellar vesicles (GUVs, ∼5.8 ± 0.6 µm) produced by the papyrus gel method. Vesicle sizes were confirmed by DLS, nanoparticle tracking analysis, or cell counter (Figure S3). Notably, the GUVs are comparable in size to human erythrocytes (∼7–8 µm). For leakage assays, we normalized lipid content across samples by adding equal total vesicle surface area per well (approximately 4.5 × 10⁹ particles for small vesicles and 9 × 10^3^ GUVs per well). Carboxyfluorescein release from vesicles was then monitored over 30 min after peptide addition.

**FIGURE 7:**
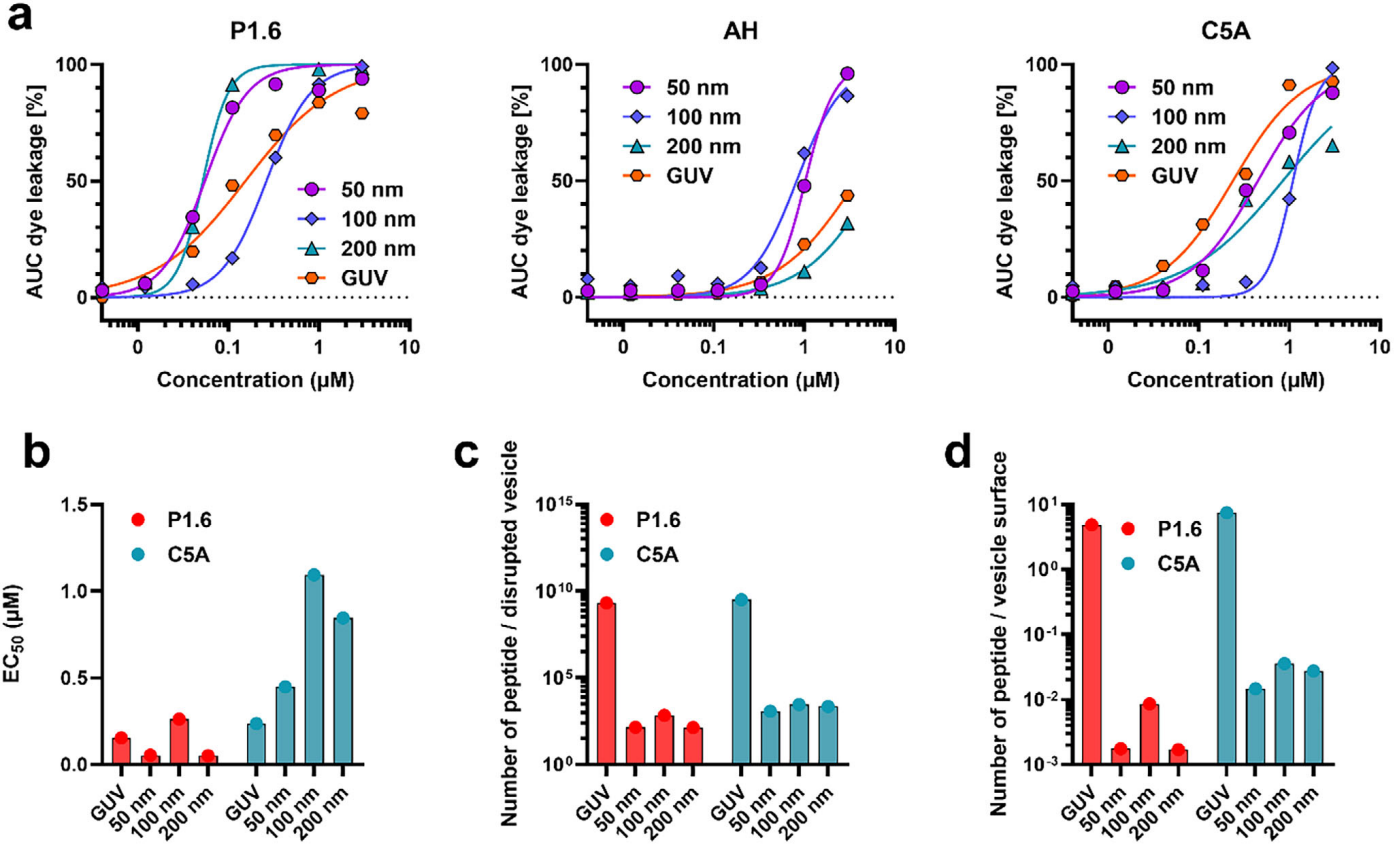
Size‐dependent membrane disruption by P1.6 compared to AH and C5A. (a) Concentration‐dependent leakage of carboxyfluorescein from liposomes of defined sizes (50, 100, 200 nm, and GUVs) upon incubation with P1.6, AH, or C5A for 30 min. Vesicles were standardized to 4.5 × 10⁹ particles/mL for liposomes and 9 × 10^3^ particles/ml for GUVs. Fluorescence intensity was measured over time and normalized to total lysis induced by Triton X‐100. Data are shown as the area under the curve (AUC). (b) EC_50_ values for membrane disruption by P1.6 and C5A, derived from the leakage data in (a). (c) Estimated number of peptide molecules required to disrupt a single vesicle at EC_50_ concentrations. (d) Peptide efficiency normalized to vesicle surface area, reflecting curvature‐dependent activity. Data represent mean values from one experiment performed in triplicate.

P1.6 disrupted membranes of all tested size classes with high potency. EC_50_ values ranged from ∼0.05 µm for 50 nm vesicles to ∼0.15 µm for GUVs, indicating that in terms of concentration the lytic activity of P1.6 was largely size‐independent (Figure 7a,b; Table 2). In contrast, AH displayed a strong dependence on vesicle size: it was active on the smallest liposomes (EC_50_ ∼1.03 µm at 50 nm) but lost activity as vesicle diameter increased (no measurable lysis of 200 nm or larger vesicles at relevant concentrations). C5A showed moderate, more uniform potency across all sizes (EC_50_ ∼0.23–1.09 µm). Despite a similar EC_50_ of P1.6 across sizes, further analysis revealed a striking efficiency advantage on highly curved membranes. On a per‐particle basis, P1.6 was ∼1.4 × 10^7 times more effective at lysing 50 nm vesicles than GUVs (Figure 7c). Even when normalized to the vastly different surface areas of these vesicles, P1.6 remained about 2.8 × 10^3 times more efficient on the 50 nm liposomes (Figure 7d). By comparison, the lytic efficiency of C5A was more consistent and only modestly influenced by size, whereas the effectiveness of AH dropped precipitously as vesicle curvature decreased. These observations indicate that P1.6 retains the ability to disrupt even very large membranes, but it is vastly more efficient on highly curved, small vesicles, likely because it preferentially targets the lipid packing defects that are more abundant on curved membranes.

**TABLE 2:** EC_50_ values and peptide‐to‐lipid (P:L) ratios for membrane disruption by P1.6, AH, and C5A across vesicle sizes.

	P1.6		AH		C5A	
	EC_50_ [µm]	P:L Ratio	EC_50_ [µm]	P:L Ratio	EC_50_ [µm]	P:L Ratio
50 nm	0.05	714	1.03	35	0.45	79
100 nm	0.26	288	0.82	91	1.09	69
200 nm	0.05	2143	—	—	0.85	126
GUV	0.15	1188	—	—	0.23	775

EC_50_ values (in µm) were derived from fluorescence‐based leakage assays shown in Figure 5a, based on the area under the curve (AUC) of dye release over time. P:L ratios were calculated from EC_50_ concentrations and the corresponding lipid content per well. “–” indicates that an EC_50_ could not be determined within the tested concentration range.

### 2.7 P1.6 Activity Depends on Membrane Composition

**FIGURE 8:**
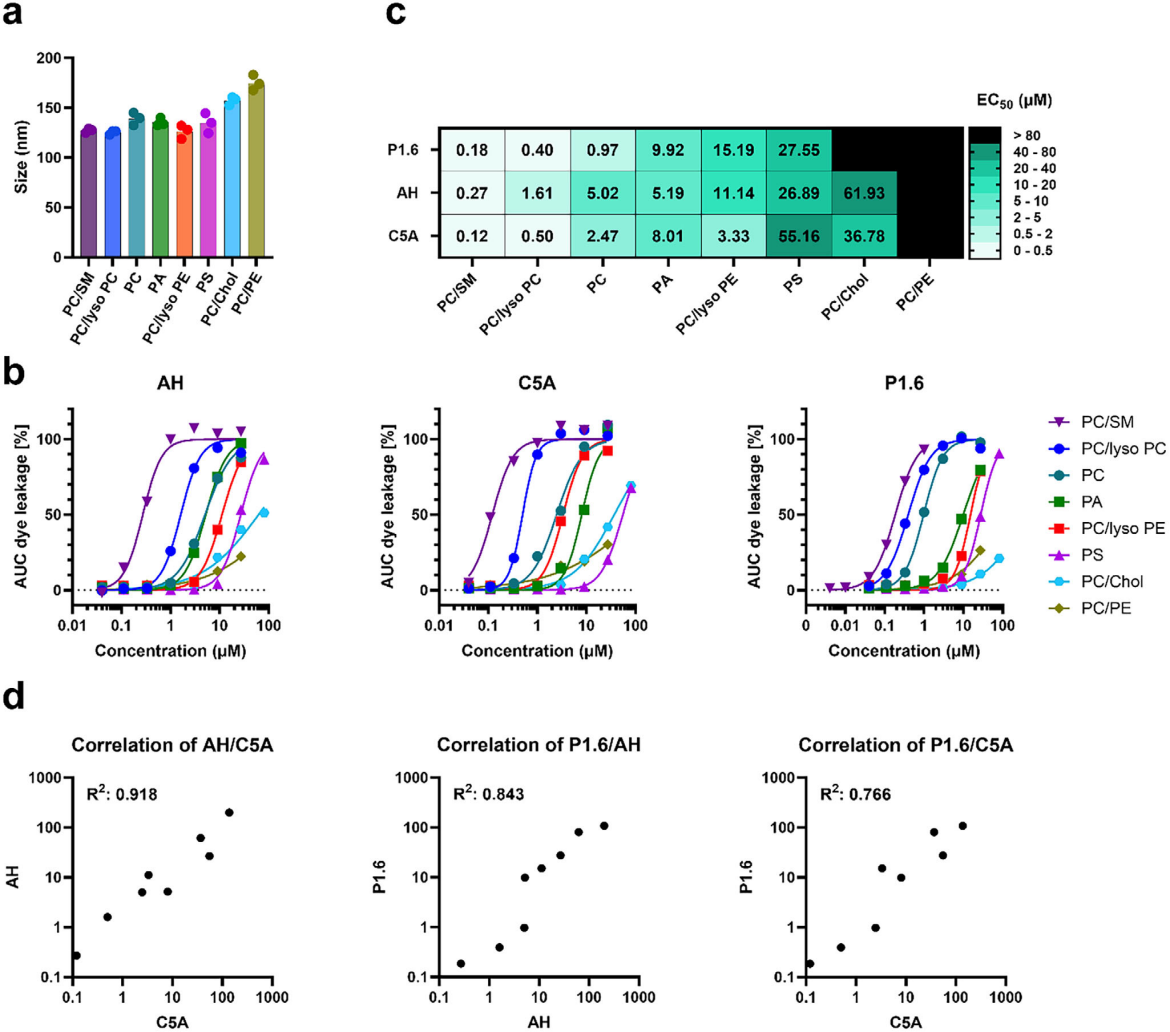
Membrane‐disruptive activity of P1.6 depends on lipid composition. (a) Mean diameters of liposomes composed of different binary lipid mixtures, as determined by dynamic light scattering. (b) Liposomes with the indicated lipid compositions were incubated with increasing concentrations of P1.6, AH, or C5A for 30 min. Membrane disruption was quantified by fluorescein leakage and is shown as the area under the curve (AUC) after baseline subtraction. Data represent mean ± SD of one experiment performed in triplicate. (c) EC_50_ values for P1.6, AH, and C5A derived from leakage assays shown in (b). (d) Correlation of EC_50_ values across lipid compositions for each peptide pair, reflecting similarity in lipid selectivity profiles.

Although vesicle size per se had little effect on the concentration of P1.6 needed for membrane disruption, the differing virus susceptibilities and the peptide's negligible hemolysis suggested that lipid composition plays a critical role in its activity. We therefore examined P1.6's lytic activity against liposomes of varying lipid makeup. A panel of liposome formulations was prepared, including those composed of phosphatidylcholine (PC), sphingomyelin (SM), lysophosphatidylcholine (lysoPC), phosphatidylethanolamine (PE), phosphatidic acid (PA), phosphatidylserine (PS), and cholesterol in various combinations. Pure SM, PE, or cholesterol liposomes could not be formed due to their biophysical properties, so these lipids were tested in mixed compositions. All liposome preparations had a total lipid concentration of 5 mm and were of similar size (on the order of 100–200 nm by NTA and DLS, Figure 8a) to ensure any activity differences were due to composition rather than vesicle size variability.

Using our fluorescein leakage assays, we compared the membrane disruption caused by P1.6, AH, and C5A across these lipid compositions. P1.6 demonstrated potent, dose‐dependent leakage of most liposome types tested (Figure 8b,c). Notably, it was highly active against zwitterionic PC/SM membranes (EC_50_ ∼0.18 µm), a profile closely matching that of AH (EC_50_ ∼0.27 µm) and C5A (EC_50_ ∼0.12 µm) on the same composition. P1.6 was similarly effective on membranes composed of PC with lysoPC, PC alone, or PC with PA, indicating a broad efficacy on fluid, predominantly neutral phospholipid bilayers. By contrast, the presence of negatively charged or order‐promoting lipids markedly reduced activity: liposomes containing 30% PS were much less susceptible to P1.6 (EC_50_ ∼27.6 µm), and cholesterol‐rich liposomes (PC/Chol) were essentially resistant to lysis by P1.6 in this assay. Importantly, none of the peptides (P1.6, AH, or C5A) induced measurable leakage in membranes high in PE (PC/PE liposomes) even at concentrations up to 80 µm, suggesting that membranes enriched in PE (a cone‐shaped lipid that can stabilize negative curvature) are inherently harder for these peptides to disrupt. A correlation analysis of EC_50_ values across all compositions revealed that P1.6, AH, and C5A share very similar lipid‐selectivity profiles (Figure 8d). Each prefers fluid, loosely packed (high‐PC/Chol–excluded) membranes and is less effective against membranes that are highly ordered or negatively charged. This suggests a common general mode of membrane disruption for all three peptides.

Given the apparent importance of cholesterol in peptide efficacy, we further investigated how increasing cholesterol content influences membrane susceptibility to P1.6. PC liposomes were prepared with 0%, 10%, 20%, 30%, 40%, and 50% cholesterol and tested in the leakage assay (Figure S4a–d). The activity of P1.6 progressively declined with rising cholesterol content (Figure S4a–c). In fact, between 40% and 50% cholesterol, there was a sharp drop in leakage, indicating that highly cholesterol‐rich membranes become largely refractory to P1.6. Cholesterol is known to increase lipid packing order and membrane rigidity at higher concentrations (30%–50%) [29], which likely hinders peptide insertion and pore formation. Consistent with this, our molecular dynamics simulations of P1.6 with POPC membranes containing 30% cholesterol demonstrate that cholesterol completely impairs P1.6's ability to bind to the membrane (Figure S9).

Our findings support this, as membranes became more rigid and tightly packed, P1.6 was increasingly unable to penetrate and disrupt them. While the hydrophobic character of P1.6 favors its initial association with membranes, excessive cholesterol likely traps the peptide at the bilayer interface without allowing the disruptive insertion. These composition‐dependent effects parallel the behavior of other membrane‐active peptides. For example, C5A is reported to have a strong affinity for cholesterol‐rich membranes [30], which might correlate with its high hemolytic activity since erythrocytes have a cholesterol concentration above 40%, mainly present in their outer leaflet, whereas the affinity of P1.6 for lower cholesterol contents is consistent with its negligible hemolysis [31, 32].

### 2.8 Mechanistic Insights about Pore Stabilization from Simulations

**FIGURE 9:**
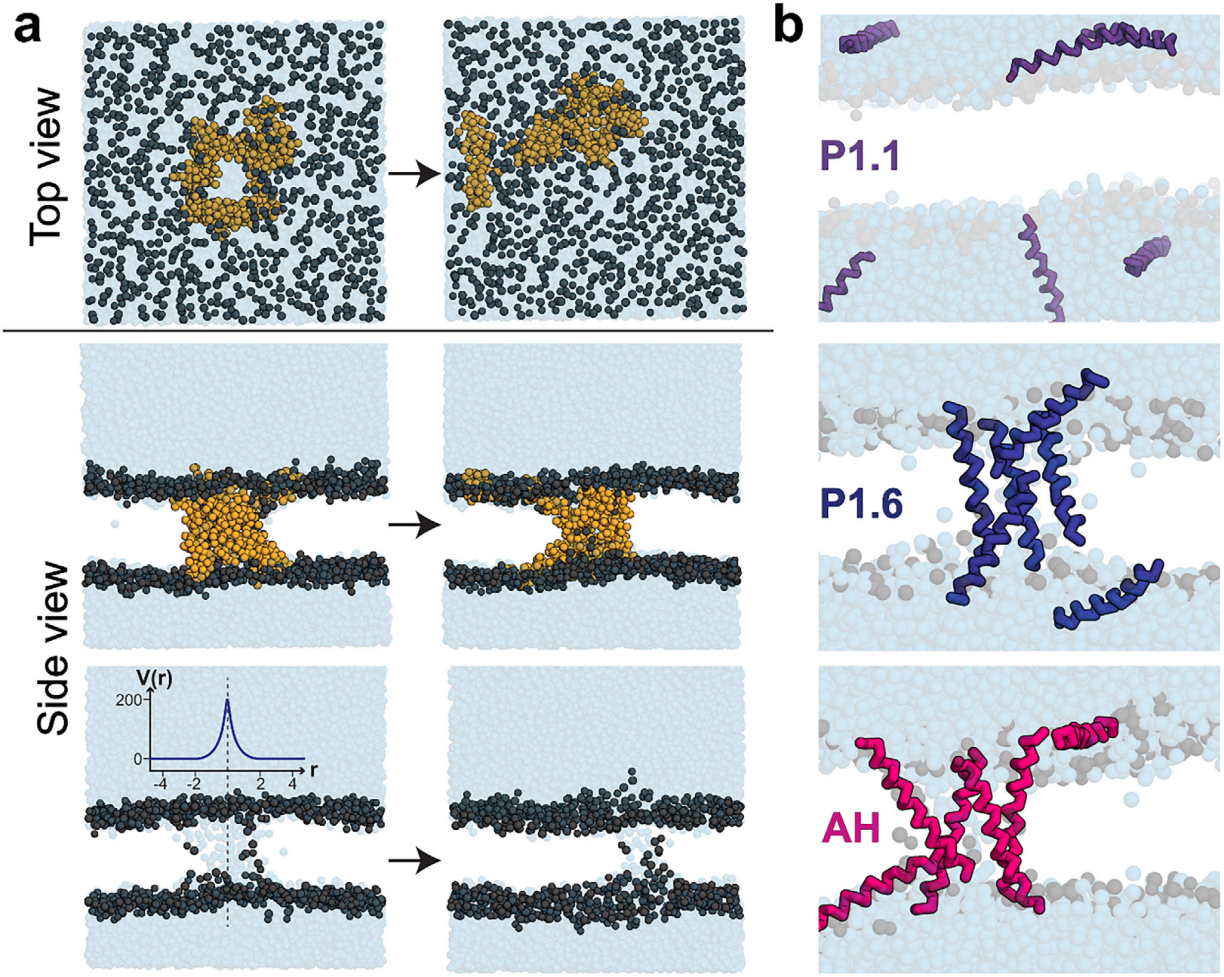
MD simulation of peptide‐induced pore stabilization. (a) Coarse‐grained MD simulations showing top and side views of a pre‐formed aqueous pore (highlighted by the FB potential) in the presence and absence of peptides. Upon removal of the FB potential, the pore collapses unless stabilized by P1.6 or AH. (b) Structural organization of peptides within the membrane: P1.1 (top) is attached to the membrane, P1.6 forms a 4/5 mer pore, and AH assembles into a four‐helix pore. Peptides are shown as ribbons colored by type; lipids are shown in gray; water molecules are omitted for clarity.

To elucidate the mechanism by which P1.6 disrupts membranes, we performed coarse‐grained molecular dynamics (MD) simulations to see whether P1.6 can stabilize transient membrane pores. In these simulations, a stable aqueous pore was first created in a lipid bilayer using a flat‐bottom restraint potential (Figure 9a, left panel). Upon removal of this restraint in the absence of peptide, the pore rapidly collapsed and the membrane resealed (Figure 9a, right panel). By contrast, when either P1.6 or the HCV‐derived AH peptide was present in the system, the pre‐formed pore remained open after restraint release, maintaining a continuous water channel across the bilayer (Figure 9b). Thus, both P1.6 and AH were capable of preventing an incipient pore from closing.

Furthermore, MD trajectory analysis provided insight into how these peptides stabilize pores. Snapshots and density profiles showed that P1.6 and AH localized at the pore interface, inserting into the lipid headgroup region and interacting with both the lipids and surrounding water (Figure 9b). In simulations with P1.6, approximately four peptide molecules accumulated around the pore rim, forming a peptide‐lined aperture through the membrane. Similarly, AH peptides formed a pore‐spanning bundle of four helices encircling the water‐filled pore. The P1.6 assembly appeared somewhat irregular or asymmetric compared to the more uniform four‐helix ring formed by AH, but in both cases the peptide oligomers effectively prevented the pore from closing over the course of the simulation. In contrast, another designed but inactive peptide (P1.1, Table S1) that we tested mostly attached to the bilayer and failed to stabilize an open pore structure.

Together, these simulations demonstrate that P1.6, like the prototypical HCV AH peptide, can stabilize a transient membrane pore by lining and fortifying the pore edges. In the absence of such peptides, small water pores in the membrane are unstable and close almost immediately.

### 2.9 P1.6 Compromises HIV‐1 Envelope Integrity

**FIGURE 10:**
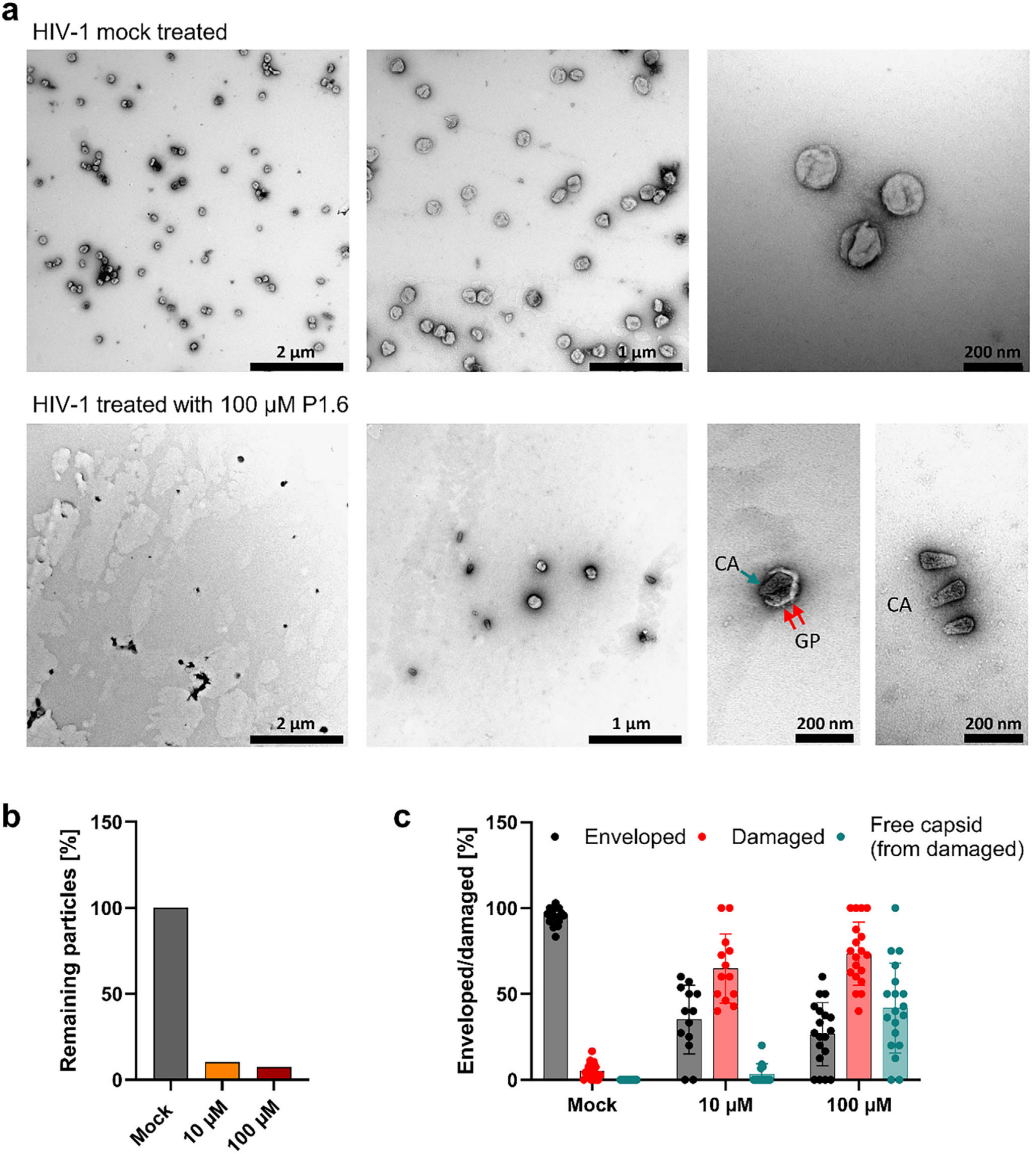
P1.6 disrupts the HIV‐1 envelope. (a) Transmission electron microscopy (TEM) images of purified HIV‐1 particles treated with PBS (mock) or 100 µm P1.6 for 30 min at 37°C, followed by fixation and negative staining with 0.5% uranyl acetate. Arrows indicate visible glycoprotein spikes (red) and free capsids (petrol). Scale bars: 2 µm (left), 1 µm (middle), and 200 nm (right). (b) Quantification of TEM images in intact and damaged virions following treatment. (c) Classification of particles into intact (enveloped), damaged, and free capsid (from damaged) categories. Data represent mean ± SD from over 550 (mock), 100 (10 µm), and 140 (100 µm) individual analyzed particles.

Finally, to determine whether the membrane‐disruptive action of P1.6 extends to actual virions, we visualized treated viruses by transmission electron microscopy (TEM). Sucrose gradient purified HIV‐1 particles (Figure S5) were incubated with P1.6 (10 or 100 µm) for 30 min at 37°C, then fixed and negatively stained for TEM imaging. Control virions treated with buffer alone (mock) appeared structurally intact, with continuous lipid envelopes, electron‐dense cone‐shaped cores (capsids), and abundant envelope glycoprotein visible on the surface (Figure 10a, top panels). By contrast, P1.6‐treated virions showed extensive envelope damage and structural disruption. Most particles had visibly breached or fragmented membranes, and numerous naked capsids (cores lacking an envelope) were observed either partially protruding from or completely released from the viral membrane remnants (Figure 10a, bottom). Quantitative analysis of hundreds of virions confirmed these observations: P1.6 exposure led to a dramatic decrease in the number of intact (enveloped) particles and a corresponding increase in damaged and fully disassembled (free capsid) particles, in a dose‐dependent manner (Figure 8b,c). At both 10 and 100 µm P1.6, over 90% of HIV‐1 particles showed some degree of envelope rupture or loss. These results demonstrate that P1.6 effectively compromises the integrity of the HIV‐1 envelope, leading to morphological disassembly that would render the virus non‐infectious. Notably, in many disrupted virions, the internal capsid remained largely intact and was simply released from the broken envelope, indicating a primarily membranolytic effect rather than non‐specific destruction of all viral components. In other words, P1.6 targets the viral membrane specifically, stripping the envelope from the virus and exposing the core, which is consistent with a mechanism of action focused on membrane disruption.

## 3 Discussion

We applied an evolutionary molecular dynamics (Evo‐MD) design strategy to successfully generate a novel amphipathic peptide, P1.6, that targets and disrupts viral envelopes—a conserved structure critical for enveloped virus infectivity. P1.6 demonstrated potent broad‐spectrum antiviral activity, notably against herpes simplex viruses (HSV‐1 IC_50_ ∼0.18 µm; HSV‐2 IC_50_ ∼0.06 µm) and Zika virus (ZIKV IC_50_ ∼0.28 µm). Importantly, it achieved these effects without detectable cytotoxicity or hemolysis at active concentrations, indicating a high selectivity for viral membranes. In direct comparisons with benchmark antimicrobial peptides (AH and C5A), P1.6 showed superior potency against ZIKV while being less effective against HIV‐1, underscoring variability in susceptibility among different enveloped viruses. These findings highlight the success of the Evo‐MD approach in yielding a potent antiviral lead (P1.6) and illustrate the importance of viral context in determining peptide efficacy.

### 3.1 Envelope Composition and Selectivity

A key factor influencing the spectrum of P1.6's activity is the lipid composition of the viral envelope. We observed that P1.6 was notably ineffective against certain enveloped respiratory viruses (hCoV‐229E, hCoV‐OC43, SARS‐CoV‐2, and IAV) that proved resistant even at high peptide concentrations. These viruses typically bud from host membranes enriched in cholesterol and sphingolipids, forming lipid raft microdomains [33, 34]. Such raft‐derived viral envelopes have exceptionally high cholesterol content and more ordered lipid domains, which significantly increase membrane stiffness and reduce packing defects [29, 33]. Our molecular dynamics simulations of P1.6 with POPC membranes containing 30% cholesterol revealed the remarkable ability of cholesterol to completely impair membrane binding. Consistent with this, our liposome experiments demonstrated that the membranolytic activity of P1.6 is markedly reduced in cholesterol‐rich membranes, whereas it remains high in sphingomyelin‐ or phosphatidylcholine‐rich membranes. In fact, P1.6, AH, and C5A all share a preference for fluid, less‐ordered lipid environments and struggle to disrupt highly cholesterol‐ or PE‐enriched bilayers.

Notably, differences in lipid selectivity translate into distinct safety profiles for these peptides. C5A, for example, has the strongest affinity for cholesterol‐rich membranes in the tested set, which correlates with significant hemolytic activity, whereas the reduced cholesterol affinity of P1.6 corresponds with the absence of hemolysis and cytotoxicity in the tested human cell lines. Thus, while the selectivity of P1.6 for certain lipid compositions confers a favorable therapeutic index, it also means that viruses with “raft‐like” cholesterol‐heavy envelopes are inherently less susceptible to it. In summary, envelope lipid composition is a major determinant of P1.6 efficacy. It helps explain the peptide's excellent activity against many viruses by targeting relatively disordered viral membranes as well as its limited effect on cholesterol‐rich viral envelopes, aligning with the observed differences between P1.6 and existing peptides like AH and C5A in both antiviral spectrum and host cell safety.

### 3.2 Glycoprotein Shielding and Membrane Accessibility

Lipid composition alone, however, cannot fully account for the variability in the antiviral effects of P1.6. Another crucial factor is the physical accessibility of the viral membrane. Many enveloped viruses, notably influenza A and coronaviruses, are densely covered with glycoprotein spikes that may form a protective shield over the viral membrane [35, 36]. This glycoprotein shielding can physically impede membrane active peptides from inserting into the lipid bilayer, thereby reducing their efficacy even when the underlying membrane would, in principle, be susceptible. In addition, the presence of viral glycoproteins substantially alters the physical properties of viral envelopes, increasing membrane stiffness [37]. Such enhanced stiffness likely reduces the susceptibility of viral membranes to the lytic action of membrane‐targeting peptides, analogous to the stabilizing effect observed for membranes with high cholesterol content. Indeed, the pronounced resistance of hCoV‐229E, hCoV‐OC43, SARS‐CoV‐2, and IAV to P1.6 and the potential need for much higher peptide concentrations to inactivate these viruses can be partly attributed to this barrier. In essence, even if P1.6 is capable of disrupting a particular lipid membrane, a virus whose envelope is tightly cloaked by glycoproteins may evade or delay peptide‐mediated lysis. It is also worth noting that experimental factors can play a role. Virus preparations often contain heterogeneous particles such as non‐infectious virions, microvesicles, and cell debris that can sequester membrane‐active peptides [38], potentially underestimating the peptide's true potency against the infectious virions.

### 3.3 Influence of Virion Size and Curvature

Interestingly, a high glycoprotein density on the virion does not invariably predict resistance to P1.6. ZIKV particles (∼50 nm in diameter) are small and densely decorated with E glycoproteins forming a tight outer surface [39], yet P1.6 was highly potent against ZIKV. One explanation is that the extreme curvature of smaller virions inherently creates lipid packing defects and transient exposure of the membrane. The dynamic arrangement or “breathing” of glycoproteins might intermittently expose patches of the lipid envelope [40], allowing P1.6 to insert despite the glycoprotein coat. Moreover, a strongly curved membrane has less ordered lipid packing and higher intrinsic strain, making it more vulnerable to disruption by amphipathic peptides [41, 42]. HSV‐1 and HSV‐2, although larger (∼150–200 nm), may likewise have envelope features that improve peptide access—for instance, a patchier distribution of glycoproteins or the presence of a tegument layer that creates local membrane vulnerabilities. These factors could allow P1.6 to penetrate or destabilize HSV envelopes more effectively than in viruses like influenza or coronaviruses, whose larger, relatively planar envelopes are uniformly coated with glycoproteins. Together, these observations emphasize that virion size and architecture (curvature, glycoprotein organization, etc.) critically influence how well a membrane‐targeting peptide can attack a given virus.

### 3.4 Mechanistic Insights into Membrane Disruption

Biophysical and imaging data lend further support to a primarily membranolytic mechanism of action for P1.6. Fourier‐transform infrared (FTIR) spectroscopy confirmed that P1.6 is largely α‐helical in structure and becomes even more helical upon binding to membranes, a common feature of many membrane‐lytic peptides [43]. This helical conformation is crucial, as it facilitates the insertion of the peptide into lipid bilayers and promotes peptide aggregation within the membrane, thereby perturbing the bilayer's integrity [44]. In addition, short N‐terminal segments may exert capping effects that stabilize local helical conformations, as described for the NKP motif in multifunctional coil/N‐cap/α‐helix scaffolds [45]. Such effects could also contribute to the structural flexibility observed in our peptides. Transmission electron microscopy provided direct visual evidence of the effect of P1.6 on actual virions, revealing “stripped” HIV‐1 particles lacking their envelopes after peptide treatment. Such images are emblematic of a membranolytic mode of action, wherein the peptide compromises the integrity of the viral envelope and causes catastrophic loss of the membrane, thereby abolishing infectivity. This outcome is analogous to how alcohol‐based disinfectants inactivate enveloped viruses by solvating their lipid envelopes.

However, in the case of P1.6, membrane disruption is achieved by a peptide that, by design, preferentially targets viral envelopes over host cell membranes. Moreover, the membrane‐focused mechanism inherently limits the potential for genetically encoded resistance: because the viral envelope is host‐derived rather than encoded by the viral genome, classical resistance through point mutations is unlikely, making mutation‐based escape improbable [46]. Although viruses could theoretically bud from alternative membrane regions, such shifts are not genetically controlled in a way that would allow systematic resistance testing.

### 3.5 Pore Formation Versus Interfacial Carpet Mechanism

An important mechanistic question is whether P1.6 disrupts membranes by forming discrete transmembrane pores or via a more distributed, carpet‐like mechanism at the membrane interface. Our coarse‐grained MD simulations indicated that P1.6 can stabilize transient membrane pores by oligomerizing at defect sites, a behavior reminiscent of the AH peptide [47]. However, functional assays of membrane leakage suggest that stable pore formation is not the primary mechanism under typical conditions. In dye‐release experiments, P1.6 induced relatively slow and incomplete leakage at lower, sub‐lytic concentrations. At higher peptide concentrations, leakage became more pronounced but remained slower compared to classical pore‐forming peptides. Notably, even a single nanometer‐scale pore would be expected to induce near‐instantaneous and complete content release from a liposome [10]. The absence of such rapid, all‐or‐none leakage in our experiments, despite ample peptide being present, suggests that long‐lived, large pores are infrequent or transient in the presence of P1.6.

### 3.6 Mechanism, Selectivity, and Comparisons

The observed activity of P1.6 at very low peptide‐to‐lipid ratios (∼1:1000–1:2000) suggests high potency and, together with molecular dynamics simulations, raises the possibility of pore formation. However, the delayed onset and incomplete leakage at these concentrations support an interfacial disruption mechanism rather than the formation of stable transmembrane pores. We propose that P1.6 operates through a context‐dependent mechanism, modulated by factors such as P:L ratio, peptide–peptide interactions, membrane composition, and vesicle size. This continuum of activity is exemplified by melittin, which exhibits rapid pore‐like leakage at low surface densities but transitions to a surface‐bound, non‐lytic state as the membrane becomes saturated with peptide [48]. Our findings suggest that P1.6 behaves similarly, capable of transient pore formation under certain conditions, yet predominantly acting through shallow membrane insertion and lipid packing disruption at concentrations relevant to antiviral activity. This interfacial mode of action aligns with its high selectivity and low host toxicity: P1.6 preferentially targets small, highly curved viral envelopes enriched in packing defects, while sparing the more ordered, less deformable membranes of host cells. In contrast, C5A requires higher concentrations for antiviral activity and exhibits significant hemolytic effects, indicative of a more detergent‐like, carpet‐model mechanism that disrupts membranes indiscriminately [43]. By comparing P1.6 with AH and C5A, it becomes evident that P1.6 occupies a favourable intermediate position, potent enough to dismantle viral membranes, yet sufficiently selective to avoid damaging host cells, owing to a mechanism finely tuned to exploit biophysical differences between viral and cellular membranes.

### 3.7 Conclusions and Future Directions

In conclusion, our work demonstrates that in silico evolution‐guided design (Evo‐MD) can yield efficacious and selective membrane‐targeting antivirals, as exemplified by P1.6, but it also underscores the need to tailor such peptides to the specific biophysical context of different viruses. The differential activities of P1.6, AH, and C5A against various enveloped viruses arise from a complex interplay of factors: the lipid composition and curvature of the viral envelope, the degree of glycoprotein shielding, and the peptide's mode of membrane interaction. By identifying these factors, we not only explain the current spectrum of P1.6 but also gain insights into how next‐generation peptides might be engineered for broader and more reliable antiviral coverage.

Building on these insights, several strategies could further improve the antiviral spectrum and therapeutic potential of P1.6. Peptide modifications are a promising avenue; for instance, conjugating P1.6 to virus‐targeting ligands (such as antibodies or receptor‐binding domains) might help deliver it to glycoprotein‐rich viral surfaces, or incorporating specific amino acid substitutions could increase its ability to insert into cholesterol‐heavy membranes. Such modifications aim to enhance efficacy against the cholesterol‐rich or heavily glycosylated viruses that currently show resistance. Advanced computational modeling will be valuable to guide these efforts. Future simulations that incorporate more realistic viral envelope models (e.g., lipid compositions with high cholesterol or sphingomyelin content, asymmetric lipid distributions, and even explicit glycoprotein lattices on the membrane surface) can provide predictive insight into how P1.6 and its analogues behave in the native virus environment. These in silico studies, coupled with biophysical experiments, could identify sequence modifications or design principles that enable peptides to overcome the stabilizing effects of rigid lipid domains or penetrate glycoprotein shields.

In parallel, deeper experimental investigation into the interplay of peptide assembly and membrane geometry could inform new designs to overcome glycoprotein barriers. For example, studies might examine whether P1.6 or derivatives can laterally diffuse under a glycoprotein layer or preferentially target membrane regions of high curvature or tension on intact virions. Insights from such studies could lead to peptides with improved ability to find and bind exposed patches of the viral membrane, even when much of the surface is protein‐coated. Additionally, optimizing the pharmaceutical properties of P1.6 will be important for clinical development. This includes improving its solubility and serum stability and fine‐tuning its specificity to avoid off‐target effects. Encouragingly, the absence of cytotoxicity and hemolysis observed for P1.6 suggests a good starting point for further optimization toward therapeutic use. Ultimately, synthetic membrane‐targeting antiviral peptides like P1.6 represent a promising new class of broad‐spectrum antivirals that exploit a fundamental vulnerability shared by enveloped viruses, their dependence on a fragile lipid envelope. By refining such peptides for greater potency and breadth while retaining selectivity, we can develop versatile countermeasures against both current and emerging viral threats. These peptides would fill an important gap in our antiviral arsenal, complementing traditional antivirals that target virus‐specific proteins with a strategy that remains effective even as viruses evolve and diversify. The present study provides a proof of concept for this approach, highlighting P1.6 and the Evo‐MD design platform as a foundation for next‐generation antivirals targeting the Achilles’ heel of enveloped viruses.

## 4 Material and Methods

### 4.1 Evolutionary Molecular Dynamics

The Evo‐MD was employed as described previously [18, 49]. The fitness guiding the evolution (Figure S7) was based on our preliminary membrane thickness gradient approach method [21]. Every population contained 512 peptides. 4 fitness elites were copied to the next iteration, as well as all peptides that were rerun at least 4 times (rerun elites). The 24 fittest peptides were selected as parents. A new population was derived from these parents through crossover, point mutations, insertions, deletions, and swapping (see ‘genetic operations’ section below). To increase convergence, the likelihoods of these respective operations were tuned after 20 iterations and 40 iterations.

#### 4.1.1 Peptide Generation

Initial peptide sequences were randomly generated as repeated (r ∈ {1,2,3,…12}) blocks of length L_block_ ∈ {2,3,4,…12}, such that the total length (r · L_block_) ranged from 2 to 24 amino acids (AAs). To reduce the search space, we only used AAs with unique physicochemical properties (bead types) within the Martini 2 model [50] and that are common in α‐helical peptides (A, E, F, K, L, M, Q, S, W, Y) [51]

#### 4.1.2 Genetic Operations

We implemented seven types of genetic operations to allow for variation in the peptide sequence, repeat number r, and block length L_block_. Every genetic operation has its own probability that was varied in the three phases of our Evo‐MD optimization (Table S2).

1. Crossover With a probability P_c_, two ‘parent’ sequences‐ a and b‐ are both split at random positions into a_1_ and a_2_, and b_1_ and b_2_. ‘Child’ one (c_1_) is a merger of a_1_ and b_2_, and ‘child’ two (c_2_) of b_1_ and a_2_. If c_1_ or c_2_ is longer than the parent sequence a or b, respectively, the sequence is truncated from the C‐terminal side to match the parent's length. If c_1_ or c_2_ is shorter than the parent sequence a or b, the sequence is completed by adding the next AAs from the latter parental segment (b_2_ for c_1_ and a_2_ for c_2_) to the C‐terminal side until it matches the parent's length.
2. Point mutation. Every position has an equal probability to be mutated, such that for maximal sequence length (here, 24), the expected number of mutations is P_m_.
3. Deletion. With a probability P_d_, one AA is randomly deleted from the sequence.
4. Insertion With a probability P_i_, one AA is randomly inserted into the sequence.
5. Charge correction: When the net charge of a peptide exceeds a set upper or lower limit, charged AAs (here, E and K) are mutated randomly until these limits are obeyed.
6. Swapping With a probability P_s_, two AAs are selected and swapped at random.
7. Repeat number mutation with a probability P_r_; the number of repeats that is applied to the sequence block randomly increases or decreases by 1.

#### 4.1.3 System Setup and Simulation

For every peptide, the fitness value is calculated from a coarse‐grained MD simulation with the Martini force field (version 2.2) [50, 52]. Lipid packing defects were modeled by applying an external thinning force to a pure POPC membrane (440 lipids, 17 × 8.5 nm2) [21]. This force compresses the middle of the membrane (x = ½ x_box_) to a thickness of approx. 3 nm and gradually relaxes to its equilibrium thickness (approx. 4 nm) at the periodic boundaries (x = 0 and x = x_box_). For efficiency, every system contains two different, independent peptides that are placed on the surface of opposing membrane leaflets in the steepest section of the defect gradient (at x = 1/3 x_box_ and at x = 2/3 x_box_), see Figure S7. The membrane's surface tension was coupled to 30.22 mN/m with the Berendsen barostat [53] to counteract the tension introduced by the thinning protocol and maintain a constant membrane area, as calibrated previously [21]. A 500‐step energy minimization and a 1.5 ps relaxation stochastic dynamics (SD) run (time step of 0.05 fs) were performed before every 500 ns MD production run (discarding the first 50 ns for equilibration).

#### 4.1.4 Fitness Calculation

A peptide's fitness value is given by the sorting force along the packing defect gradient. This force was calculated by measuring the x‐component of the deviation in the distance between the peptide and a POPC choline bead (restrained in the x‐dimension, free in y and z) in the opposite membrane leaflet (Figure S7a,b) One could imagine this as a spring with spring constant k = 50 kJ mol^−1^ nm^−2^ and equilibrium length x_0_ between the peptide's center‐of‐mass and the POPC head group. The force exerted on the peptide by this spring is given by F = k(x − x_0_). This force measures the effective attraction toward (F < 0) or repulsion from (F > 0) the thinned region. The more negative’ this force, the ‘fitter’ the peptide.

#### 4.1.5 Peptide Synthesis and Purification

All peptides used in this study were synthesized by Dennis Aschmann and Alexander Kros and by Synpeptide (China) at a purity of >95% using standard Fmoc‐based solid‐phase peptide synthesis. Peptides were purified to a final purity of >95% using reverse‐phase high‐performance liquid chromatography (RP‐HPLC). The molecular masses of the purified peptides were verified via matrix‐assisted laser desorption/ionization time‐of‐flight mass spectrometry (MALDI‐TOF MS). Lyophilized peptide samples were stored at −20°C to maintain stability during long‐term storage. For in vitro experiments, peptide aliquots were prepared by initially dissolving lyophilized peptides in 100% dimethylformamide (DMF) to ensure complete solubilization. The dissolved peptides were subsequently diluted in ultrapure water at room temperature to obtain stock solutions with a final concentration of 4 mm in 10% DMF. These stock solutions were stored at −20°C until further use. Prior to experimental applications, peptides were pre‐diluted in ultrapure water and titrated into the respective experimental media, such as phosphate‐buffered saline (PBS) or cell culture medium, depending on assay requirements. Control peptides AH and C5A were obtained from Synpeptide (China) at a purity of >95%. These peptides were synthesized as described above and verified via MALDI‐TOF MS. The sequences used for AH and C5A peptides were from N→C SGSWLRDVWDWICTVLTDFKTWLQSKL‐NH2 for AH and SWLRDIWDWICEVLSDFK‐NH2 for C5A taken from Jackman et al. 2018 [54] and Veazey et al. 2016 [55]. Unlike experimental peptides, control peptides were dissolved in dimethyl sulfoxide (DMSO) instead of DMF. In all in vitro experiments, the final concentration of organic solvent in peptide solutions was maintained below 0.25% to minimize solvent‐related effects.

### 4.2 All‐Atom Molecular Dynamics

#### 4.2.1 Membrane System

The peptide was generated in an initial alpha‐helical conformation using PyPept [1] and positioned near the surface of a pre‐equilibrated POPC lipid bilayer. The system comprised 238 POPC lipids, one peptide, 21520 TIP3p water molecules, and 54 Na+ and 54 Cl‐ ions (corresponding to ∼150 mm NaCl).

The system was equilibrated for 500 ns, during which distance restraints were applied to the alpha carbons of the peptide to maintain the initial secondary structure. Afterward, three independent 500 ns production simulations were performed to ensure reproducibility.

#### 4.2.2 Solvent System

The peptide was generated in an initial alpha‐helical conformation using PyPept [56] and placed in a solvent box. The system comprised one peptide, 13520 TIP3p water molecules, and 36 Na+ and 36 Cl‐ ions (corresponding to ∼150 mm NaCl). A short 10 ns simulation was performed during which distance restraints were applied to the alpha carbons of the peptide to maintain the initial secondary structure. Afterward, three independent 1000 ns production simulations were performed to ensure reproducibility.

#### 4.2.3 Simulations

All‐atom molecular dynamics (MD) simulations were performed using the GROMACS 2023.5 simulation package using the CHARMM36m force field (July 2022 release). Temperature was maintained at 293 K using the velocity‐rescale thermostat (tau = 0.5 ps), applied separately to the peptide, lipids, and solvent groups. Pressure was maintained at 1 bar with semi‐isotropic pressure coupling using the C‐rescale barostat (tau = 5.0 ps). Simulations were carried out with a 2 fs timestep using a leap‐frog integrator. All bonds involving hydrogens were constrained using the LINCS algorithm. Long‐range electrostatics were computed with the Particle‐Mesh Ewald (PME) method with a real‐space cuttoff of 1.2 nm. Van der Waals interactions were smoothly switched to zero between 1.0 and 1.2 nm using a force‐switch function, and the Verlet cutoff scheme was used.

#### 4.2.4 Analysis

The DSSP algorithm was used for secondary structure prediction as implemented by the GROMACS 2023.5 “gmx dssp” tool. Hydrogen bond occupancies were computed using the GROMACS 2025.2 “gmx hbond” tool (@ NOTE the newer version, this is a different tool than in the GROMACS 2023.5 version). For the hydrogen bond occupancies, the first 250 ns of each simulation trajectory was not taken into account to allow the initial structure to relax.

### 4.3 Viability Assays

TZM‐bl cells (RRID: CVCL_B478) were seeded at a density of 10 000 cells, Vero E6 cells (RRID: CVCL_0574) at 6000 cells, ELVIS cells [57] at 5000 cells, CaCo‐2 cells (RRID: CVCL_0025) at 20 000 cells, and Huh7 (RRID: CVCL_0336) cells at 25 000 cells per well, each in 100 µL of culture medium (all cells have been tested for mycoplasma contamination on a regular basis). Plates were incubated at 37°C with 5% CO_2_ overnight to allow cell adherence. Cell conditions were in line with the associated virus assays. Compounds were diluted to a final concentration of 20 µm on cells, serially diluted 1:3, and added to the cells. A DMF control was prepared in the same manner, resulting in a maximal concentration of 0.05% on cells. At 3 days post‐treatment (dpt) for TZM‐bl and Huh7 cells and 2 dpt for Vero E6, CaCo‐2, and ELVIS cells, cells were visually inspected under a microscope to assess morphology changes or toxicity. For viability measurement, the CellTiter‐Glo assay was used. For this assay, the supernatant was removed from each well before adding the reagent. The prepared CellTiter‐Glo mix was diluted 1:1 with PBS, and 60 µL of the diluted mix was added to each well. Plates were incubated for 10 min at room temperature in the dark to stabilize the luminescence signal. After incubation, 50 µL of the solution was transferred into white plates for luminescence measurement. Samples were measured using an Orion II Microplate Luminometer (Titertek Berthold) with a measuring time of 0.1 s per well.

### 4.4 Hemolysis Assay

Five milliliters of whole blood from three donors were collected. Human blood was obtained with informed consent under approval number 157/27 from the Ethics Committee of Ulm University. The samples were centrifuged for 10 min at 1000 g (2200 rpm) at 4°C. Blood serum was removed, and erythrocytes were serially diluted. Cell size and number were measured using a Luna II Cell Counter. The erythrocyte suspension was diluted 1:40, and 40 µL per well was transferred into a V‐well plate. Compounds P1.6 and controls AH and C5A were serially diluted 1:2 in PBS in a U‐well plate, with the highest concentration set to 200 µm on cells. Ten microliters of the peptide titration were added to the cells, and 1% Triton X‐100 was included as a positive control. Samples were incubated at room temperature for 1 or 24 h under gentle orbital shaking at 500 rpm. Following incubation, the plates were centrifuged for 5 min at 1500 rpm. After centrifugation, 35 µL of the supernatant was carefully transferred to a flat‐well plate, avoiding the pellet. Plates were short‐spun (max 2000 rpm) to ensure the removal of bubbles. The absorbance was measured at 405 nm using a plate reader.

### 4.5 Infection Assays

#### 4.5.1 HIV‐Infection Assay

The adherent TZM‐bl reporter cell line was obtained from NIH. The cell line was split regularly twice a week. Cultivation was performed in DMEM (10% inactivated fetal calf serum (FCS) (Gibco, Life Technologies, Frederick, MD), 1% Penicillin/Streptomycin (Thermo Fisher Scientific), 1% Glutamine (Thermo Fisher Scientific). Virus Stocks of R5‐tropic HIV‐1 were prepared by transient transfection of HEK‐293T cells as described in von Maltitz et al. 2024. 10k TZM‐bl cells have been seeded in a 96‐wellplate and settled overnight. Virus was titrated prior, and the dilution for an infectivity of 50k RLU was determined. Peptide dilution was prepared to result in a maximum concentration of 100 µm on the virus. For the virus treatment virus was diluted in serum‐free media and mixed 1:1 with peptide titrated 1:3 in PBS. The peptide and virus have been incubated for 30 min at 37°C, and the peptide virus mix was added 1:10 on the cells and incubated at 37°C. 3 days post‐infection (dpi) infection was checked at the microscope, the supernatant was discarded, and the cells were treated with a 1:4 dilution of ß‐gal substrate (GalScreen Invitrogen) in PBS containing 0.217% Triton X‐100 (Sigma‐Aldrich), resulting in a final Triton X‐100 concentration of 0.16% on cells, to ensure virus inactivation [58]. After 40 min incubation at RT, samples were measured at the Orion II Microplate reader.

#### 4.5.2 ZIKV‐Infection Assay

For the ZIKV infection assay, 6 k Vero E6 cells obtained from (American Type Culture Collection (ATCC)) were seeded on a 96‐well plate and settled overnight. ZIKV (MR766 provided by Bernhard Nocht Institute for Tropical Medicine, Hamburg, Germany) was diluted to obtain a MOI of 0.15. The compound was titrated, and cells were infected as shown above. After 2 dpi, infection was checked at the microscope, and infection intensity was measured by in‐cell ELISA [59]. Infected cells were fixed with 4% PFA for 20 min RT, followed by permeabilization with ice‐cold MeOH for 5 min. After permeabilization, the first AB (1:10.000) in diluent buffer (PBS, 10% FCS, 0.3% Tween 20) was added to the cells and incubated for 1 h at 37°C. Followed by 3 washing steps in washing buffer (PBS, 0.3% Tween20 (Company) and subsequent addition of 50 µL of HRP conjugated second antibody (1:20.000) (NAME ANTIBODY) in diluent buffer. After incubation for 1 h at 37°C, cells were washed 4 times with washing buffer. Subsequently, 50 µL TMB substrate was added to the cells and incubated for 5 min. The reaction was stopped with 0.5 M H_2_SO_4_ (SIGMA) followed by absorption measurement at 405 nm (Molecular Devices LLC).

#### 4.5.3 HSV Infection Assay

5k Elvis reporter cells (optioned from ATCC) were seeded on a 96‐well plate and settled overnight. HSV‐1/2 was diluted to an MOI of 0.6. The compound was titrated, and cells were infected as shown above for HIV‐1. After 2 dpi, infection was checked at the microscope, and infection intensity was measured via ß‐gal assay as shown above.

#### 4.5.4 HCoV Infection Assay

25 000 Huh7 (for OC43) or 20 000 Caco2 (for NL43) were seeded per well in a 96‐well plate with 100 µL of culture media. The next day, peptide stock solutions (4 mm) were diluted in PBS to a final concentration of 200 µm and titrated in a 1:3 serial dilution. Remdesivir (by Selleckchem) was prepared at 100 µm, and an equally diluted DMF control was included, treated with a resulting maximum concentration of 0.05% on cells. The peptide titration was mixed 1:1 with virus (hCoV OC43 or NL43) and incubated at 33°C for 30 min before being added to the cells (MOI 0.1). Plates were then incubated at 33°C. Three days post‐infection (DPI), cells were fixed with 4% PFA for 30 min at room temperature. Plates were removed from biosafety level 2 containment, and the supernatant was discarded. Cells were permeabilized with 100 µL of 0.1% Triton X‐100 for 5 min, followed by washing with washing buffer PBS‐T (PBS, 0.3% Tween‐20). Immunostaining was performed using a rabbit anti‐nucleocapsid antibody (BRAND) diluted 1:5000 in PBS‐T with 10% FBS, incubated at 37°C for 1 h. After washing twice with washing buffer, a secondary anti‐rabbit HRP‐conjugated antibody diluted 1:10 000 in PBS‐T with 10% FBS was added and incubated at 37°C for 1 h, followed by three washes with washing buffer. For ELISA detection, 50 µL of TMB substrate was added per well and incubated at room temperature for 5 min in the dark. The reaction was stopped by adding 50 µL of 1 M H_2_SO_4_, and absorbance was measured at 450 and 620 nm using the vMax Kinetic ELISA microplate reader (Molecular Devices).

#### 4.5.5 IVA Infection Assay

CaCo‐2 cells were seeded at a density of 20,000 cells per well in 96‐well plates with 100 µL of cDMEM (DMEM supplemented with 7.5% (v/v) BSA, 2 mm L‐glutamine, 100 U/mL penicillin, 100 µg/mL streptomycin, and 25 mm HEPES). Plates were prepared and incubated overnight at 37°C with 5% CO_2_ to allow cell adherence. Influenza A virus strain A/PR/8/34 (H1N1; PR8) was used for infection. The virus was pre‐diluted 1:10 in PBS, and a final stock for an MOI of 0.0007 was prepared for infection. Peptides P1.6 and controls AH and C5A were prepared as stock solutions at 200 µm, titrated in a 1:3 serial dilution in PBS, and an equally diluted DMF control was included, with a resulting maximum concentration of 0.05% on cells. For infection, the virus solution was mixed 1:1 with the peptide dilution and incubated for 30 min at 37°C. After incubation, the virus‐compound mixture was transferred onto the cells for infection. At 2 days post‐infection (dpi), cells were washed with PBS and lysed with 1% Triton X‐100 for 30 min at room temperature. Lysates were centrifuged at 2000 rpm for 5 min, and supernatants were transferred to fresh tubes. For analysis, lysates were mixed 1:1 in MES buffer (25 µL each), and 20 µL was transferred to black‐walled plates. Then, 30 µL of 100 µm MUNANA (2′‐(4‐methylumbelliferyl)‐N‐acetylneuraminic acid) substrate (Thermo Fischer Scientific) was added. Plates were incubated at 37°C for 4 h, shaking at 500 rpm. The reaction was stopped by adding 150 µL of stop solution (0.1 M glycine and 25% ethanol). Fluorescence was measured at an excitation/emission of 360/455 nm using a microplate reader.

#### 4.5.6 SARS‐CoV‐2 Infection Assay

CaCo‐2 cells were seeded at a density of 20 000 cells per well in 96‐well plates with 100 µL of DMEM and incubated overnight at 37°C with 5% CO_2_. SARS‐CoV‐2 Delta variant (B.1.617.2) was used for infection. Virus stocks were pre‐diluted in DMEM to a working dilution with a resulting MOI of 0.0007. P1.6 and control C5A were pre‐diluted in PBS to 180 µm and titrated in a 1:3 serial dilution in PBS. A DMF control was included and equally diluted, with a resulting maximum concentration of 0.05% on cells. Remdesivir was prepared at 25 µm. The virus dilution was mixed 1:1 with compound titration and incubated for 30 min at 37°C. The virus/compound mix was then added to the cells. Plates were incubated at 37°C with 5% CO_2_ until analysis. At 2 dpi, cells were fixed by adding 4% PFA (1:1 dilution on cells) and incubated for 30 min at room temperature. The supernatant was removed, and cells were permeabilized by adding 100 µL of 0.1% Triton X‐100 in PBS, followed by a 5 min incubation at room temperature. Wells were then washed with PBS. For immunostaining, primary antibody (anti‐nucleocapsid, 1:5000 in antibody dilution buffer) (PBS, 0.3% Tween‐20 + 10% FBS) was added and incubated at 37°C for 1 h. After two washes with washing buffer (PBS, 0.3% Tween‐20), secondary antibody (anti‐mouse HRP‐conjugated, 1:15 000 in antibody dilution buffer) was added and incubated at 37°C for 1 h. Wells were washed three times before detection. For colorimetric detection, TMB substrate was added, and the reaction was allowed to proceed for 5 min in the dark at room temperature. The reaction was stopped with 1 M H_2_SO_4_, and absorbance was measured at 450/620 nm using a microplate reader.

### 4.6 Liposome Leakage Assays

#### 4.6.1 Liposome Preparation

Liposomes were prepared using a thin‐film hydration and extrusion method. Lipids were mixed at a ratio of DOPC/SM/Chol at 45/25/30 mol% for virus‐like liposomes, while equimolar concentrations were used for heterologous liposomes, and indicated concentrations for homologous liposomes, to achieve a final concentration of 5 mm. Lipid mixtures were prepared in glass vials, and chloroform was evaporated under a nitrogen stream. The resulting lipid film was hydrated with a 50 mm isoosmolar 5(6)‐carboxyfluorescein solution in 50% PBS, adjusted to pH 7.4 with NaOH. The lipid suspension was incubated at 60°C and 160 rpm for 1 h. Liposomes were then extruded through polycarbonate membranes with pore sizes ranging from 0.05 to 0.2 µm for at least 30 cycles using a Mini Extruder heated to 60°C. Non‐encapsulated dye was removed via gravity‐based size‐exclusion chromatography using PD midiTrap Sephadex G‐25 columns, performed twice.

#### 4.6.2 Liposome Characterization

Liposome size and concentration were determined using nanoparticle tracking analysis (NTA) on a Zeta View Twin or dynamic light scattering (DLS) with a Zeta Sizer Nano. For NTA measurements, liposomes were diluted in PBS and analyzed at 25°C with 11 positions, 1 cycle, sensitivity 85–90, shutter 100, 15 fps, and 2‐second videos per position, with 3–5 replicate measurements. The measuring chamber was flushed with PBS between samples. For DLS, samples were diluted in PBS and measured in a cuvette with automated settings for attenuator and position, with three independent acquisitions per sample.

#### 4.6.3 Generation and Characterization of Giant Unilamellar Vesicles (GUVs)

GUVs were prepared following a previously published method [11]. Lipids were mixed in a glass flask at a final concentration of 5 mm and applied to Whatman Grade 1 paper placed between CellCrown24 inserts in a 24‐well plate. After chloroform evaporation under nitrogen, the lipid film was hydrated with 1.5 mL of 50 mm 5(6)‐carboxyfluorescein solution in 50% PBS, pH‐adjusted to 7.4. The plate was incubated at 60°C with 160 rpm for 1 h. GUVs were detached by pipetting and purified twice using PD midiTrap Sephadex G‐25 columns. Size and concentration were measured using a Luna II Cell Counter at three positions.

#### 4.6.4 Dye Leakage Assay

Liposome and GUV stability was assessed by a dye leakage assay. A total of 90 µL of liposomes or GUVs at defined concentrations was added to 96‐well plates. Baseline fluorescence was recorded at an excitation wavelength of 485 nm and emission at 528 nm for 5 min using a Synergy H1 plate reader (BioTek). Then, 10 µL of peptide solution at increasing concentrations was added, and fluorescence was monitored every minute for 30 min. Maximum dye release was determined by adding Triton X‐100 at a final concentration of 1%, followed by fluorescence measurement after 5 min. Background signals were subtracted, and values were normalized to Triton‐induced fluorescence. The area under the curve (AUC) was calculated and plotted for each concentration using GraphPad Prism.

### 4.7 Transmission Electron Microscopy

#### 4.7.1 Cell Transfection

HEK293T cells were seeded at a density of 8 million cells per T175 flask (n = 10) in DMEM supplemented with 10% fetal bovine serum (FBS), 1% L‐Glutamin, and 1% penicillin/streptomycin. After 24 h, TransIT‐LT1 transfection reagent (Mirus) was warmed to room temperature and vortexed gently. A total of 5 mL OptiMEM (Gibco) was placed into a 15 mL Falcon tube, and the transfection mixture, including plasmid DNA encoding the virus of interest, was prepared according to manufacturer instructions. The mixture was incubated at room temperature for 20 min before being added dropwise to the cells. The transfected cells were incubated at 37°C with 5% CO2 for 48–72 h before supernatant collection.

#### 4.7.2 Virus Purification

The culture supernatant was harvested and collected in 50 mL tubes. The supernatant was first centrifuged at 4000 rpm for 5 min to remove cellular debris. The clarified supernatant was then transferred to new 50 mL tubes. Six 38.5 mL Open‐Top Thinwall Ultra‐Clear Tubes (Beckman Coulter) were prepared with a layer of 6 mL 20% sucrose. The supernatant was carefully layered onto the sucrose and ultracentrifuged at 20 000 rpm for 2 h at 4°C using a SW32 Ti rotor (Beckman Coulter). The supernatant was discarded, and the pellet was resuspended in 150 µL PBS and incubated in a 50 mL Falcon tube for 30 min at 4°C. A discontinuous sucrose gradient was prepared in 13.2 mL Open‐Top Thinwall Ultra‐Clear Tubes (Beckman Coulter) with 14 steps ranging from 20% to 60% sucrose concentration, prepared in H_2_O. Virus pellets were pooled and carefully layered onto the gradient and ultracentrifuged at 38 000 rpm for 2 h at 4°C using a SW40 Ti rotor (Beckman Coulter). Following centrifugation, the top 1 mL was discarded, and the gradient layers were carefully transferred in 0.7 mL fractions into fresh 1.5 mL Eppendorf tubes and vortexed. A 500 µL aliquot of each suspension was added to 1.5 mL PBS in 2 mL Eppendorf tubes, vortexed thoroughly, and centrifuged at 14 000 rpm for 2 h at 4°C. The supernatant was discarded, and the final pellet was resuspended in 100 µL of 0.25 µm filtered PBS. The concentrated virus was aliquoted and stored at ‐20°C for downstream applications.

#### 4.7.3 Nanoparticle Tracking Analysis (NTA)

For particle size and concentration measurements, fraction 4–7 of the viral preparations were first diluted 1:100 in 8% paraformaldehyde (PFA) and incubated for 20 min at room temperature for inactivation. Subsequently, the virus suspension was further diluted 1:100 in PBS, and an additional 1:5 dilution in PBS was performed. The prepared samples were analyzed using a ZetaView Twin (Particle Metrix) following manufacturer settings.

#### 4.7.4 Infectivity Assay

To check the infectivity of the purified virus fractions, TZM‐bl cells were seeded at a density of 10 000 cells per well in a 96‐well plate in 100 µL of DMEM supplemented with 10% fetal bovine serum (FBS) 1% L‐Glutamin, and 1% penicillin/streptomycin. After 24 h, 10 µL of medium was removed from cells, and virus fractions were diluted 1:20 000 before 10 µL of the diluted virus was added to the cells. 2 DPI, infection rates were measured by β‐galactosidase activity in cell lysates using the Gal‐Screen system (Applied Biosystems) supplemented with 0.16% Triton‐X (Sigma #T9284). Luminescence due to substrate conversion was quantified using an Orion microplate luminometer (Berthold).

#### 4.7.5 Sample Preparation and Imaging

The virus preparation used for TEM was obtained from purification fraction Nr 5, with a final particle concentration of 2.4 × 10^12^ particles/mL. Virus was diluted to 8 × 10^11^ particles/mL and treated 1:1 with 10 and 100 µm P1.6 for 30 min in a total volume of 20 µl at 37°C Subsequently, samples were inactivated by a 1:1 addition of 8% PFA, incubated for 30 min at 37°C, and subjected to negative staining. For that, 5 µL of the prepared virus sample (final of 1 × 10^9^ Particles per grid) was applied to Formvar‐carbon‐coated copper grids and allowed to settle for 1 min at room temperature. Grids were then subjected to three sequential washes in ultrapure water, followed by immersing them subsequently into three drops of 0.5% uranyl acetate in water for negative staining. Excess liquid was blotted with filter paper, and the stained grids were air‐dried before imaging. TEM imaging was performed using a transmission electron microscope (JEM1400, Jeol) operated at 120 kV accelerating voltage, and images were recorded with a CCD camera (Veleta, Olympus).

### 4.8 Attenuated Total Reflectance Fourier‐Transform Infrared Spectroscopy (ATR‐FTIR)

#### 4.8.1 Sample Preparation

Peptide samples were prepared as described in the peptide synthesis and purification section. Lyophilized peptides P1.6 and ctrl peptide AH were initially dissolved in 100% dimethylformamide (DMF) or dimethyl sulfoxide (DMSO) and subsequently diluted in ultrapure water to obtain a final working concentration of 400 µm. All peptide solutions were stored at ‐20°C until use and thawed and vortexed immediately before experiments. DOPC lipid solutions were prepared by dissolving lipids in chloroform and evaporating the solvent under a nitrogen stream, followed by hydration in 10 mm Phosphate buffer to achieve a final lipid concentration of 400 and 4000 µm. The hydrated lipid suspensions were then sonicated for 10 min at room temperature (RT) using a bath sonicator to facilitate vesicle formation. Peptide‐lipid interactions were studied by incubating peptides with the prepared DOPC vesicle solutions at peptide‐to‐lipid molar ratios of 1:1 and 1:10. The final reaction mixtures were vortexed briefly and incubated at RT for 30 min to allow for membrane interaction. After incubation, samples were lyophilized.

#### 4.8.2 Measurement

ATR FT‐IR spectroscopy was used to analyze peptide secondary structure. ATR FT‐IR spectra of the solid sample were recorded using a Bruker Tensor 27 spectrometer equipped with a diamond crystal as an ATR element (PIKE Miracle) with a spectral resolution of 4 cm−1, 64 scans. Measurements were conducted in three technical replicates. FTIR spectral data were processed using Origin software for baseline correction and peak fitting. Secondary structure deconvolution was performed by fitting the amide I region (1600–1700 cm^−1^) using Gaussian functions. Graph visualization was performed by using GraphPad Prism. To validate peptide integrity and ensure reproducibility, control spectra of buffer solutions and untreated peptides were recorded before each experiment. Additionally, replicate measurements of each sample were performed, and spectral consistency was confirmed across independent preparations.

### 4.9 Statistical Analysis

Data were pre‐processed by background subtraction and normalization to the respective controls. No outlier processing was performed. Data are presented as mean ± SD (cell viability, liposome leakage, respiratory Virus assays, TEM quantification), and data from virus assays (ZIKV, HIV‐1, HSV‐1/2), hemolysis assays as mean ± SEM, with *n* values indicated in the figure legends. No statistical hypothesis testing was applied to these datasets. For Figure 8, correlation analysis was carried out between peptide P1.6, AH, and C5A using Pearson correlation coefficients (two‐tailed, 95% confidence interval). Analyses were performed with GraphPad Prism version 10.4.1 (GraphPad Software, San Diego, CA, USA) and OriginPro, Version 2023b. (OriginLab Corporation, Northampton, MA, USA) for FTIR Data.

## Author Contributions

P. V. M. carried out conceptualization, investigation, formal analysis, data curation, methodology, validation, and visualization, and drafted the original manuscript. N. V. H. contributed to conceptualization, methodology, and visualization. T. W. contributed to conceptualization, data curation, supervision, and methodology. T. T. carried out the investigation and methodology. J. Me. contributed visualization. D. A. and A. K. provided resources. C. R. contributed validation. J. G. contributed supervision, methodology, and validation. H. J. R. contributed funding acquisition, methodology, resources, and validation. J. M. contributed conceptualization, funding acquisition, project administration, and revised the original draft.

## Conflicts of Interest

The authors declare no conflicts of interest.

## Supporting information




**Supporting File**: advs73690‐sup‐0001‐SuppMat.docx.

## Data Availability

The data that support the findings of this study are available from the corresponding author upon reasonable request.
