# Supplementary material for: Identification and Characterization of an In Silico Designed Membrane‐Active Peptide with Antiviral Properties
Source: Adv Sci (Weinh). 2026 Jan 9;13(16):e13911. doi: 10.1002/advs.202513911 (PMC13042762; doi:10.1002/advs.202513911)
Supplement: Supplementary file 1 — Supporting File: advs73690‐sup‐0001‐SuppMat.docx. [file ADVS-13-e13911-s001.docx]

**Identification and Characterisation of an *In-Silico* Designed**

**Membrane-Active Peptide with Antiviral Properties**

Pascal von Maltitz^1^, Niek van Hilten^2^, Tatjana Weil^1^, Thunchanok Thummaraj^4^, Jeroen Methorst^3^, Dennis Aschmann^2^, Alexander Kros^2^, Clarissa Read^5^, Jasmina Gačanin^4,^ Herre Jelger Risselada^3^ and Jan Münch^1#^

^1^ Institute of Molecular Virology, Ulm University Medical Center, Ulm, Germany

^2^ Leiden Institute of Chemistry, University Leiden, Nederland’s

^3^ Faculty of Physics, Technical University Dortmund, Germany

^4^ Max Planck Institute for Polymer Research, Mainz, Germany

^5^ Central Facility for Electron Microscopy, Ulm University

# Corresponding author: jan.muench@uni-ulm.de

Supplementary Information:


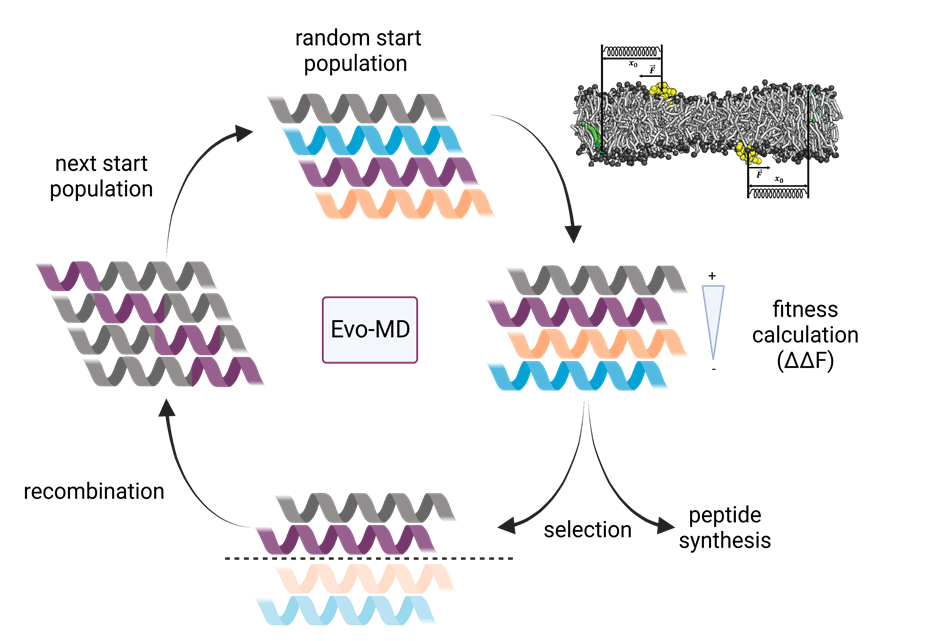


**Figure S1.** Illustration of the evolutionary molecular dynamics (Evo-MD) concept. Random peptide sequences evolve over time into lipid packing sensing and membrane disruptive peptides. Adapted from J Methorst et al, 2021 (16)


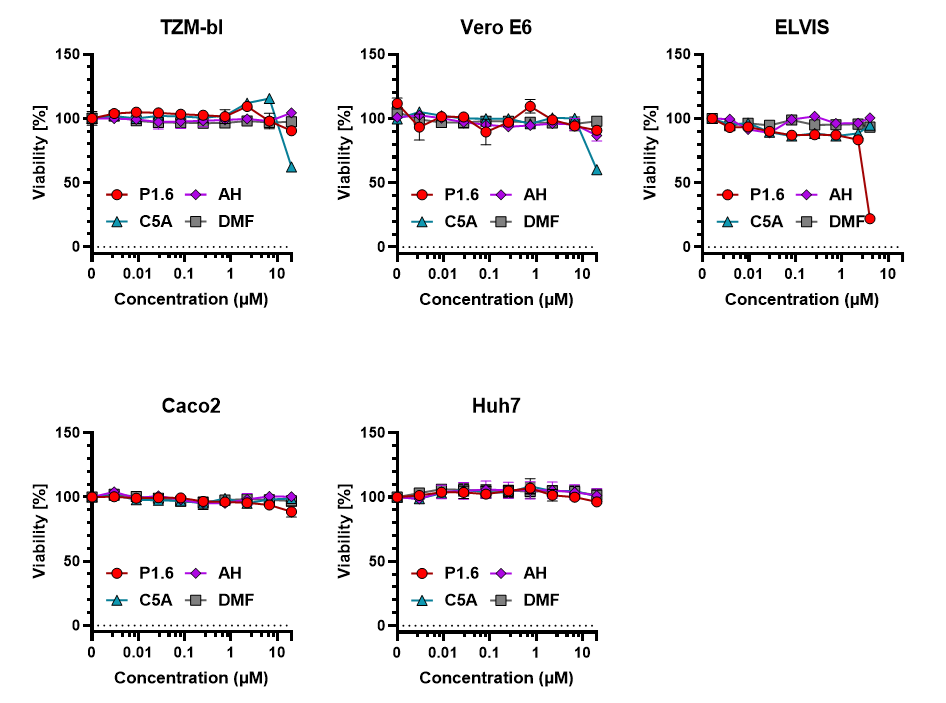


**Figure S2. Cytotoxicity of P1.6 in cell lines used for antiviral assays.** Cells corresponding to the respective virus infection models (TZM-bl, Vero E6, ELVIS, Caco2, Huh7) were treated with titrated concentrations of P1.6, AH, C5A, or vehicle control (DMF). Cell viability was assessed by quantifying intracellular ATP levels using the CellTiter-Glo Luminescent Cell Viability Assay at 3 days post-treatment (dpt) for TZM-bl and Huh7 cells, and 2 dpt for Vero E6, Caco2, and ELVIS cells. Data represent mean ± SD from 1–2 independent experiments performed in triplicates.

**Table. S1. Evo-MD predicted peptides.**

| **Peptide** | **Sequence** | **MW** | **pI** | **GRAVY** |
| --- | --- | --- | --- | --- |
| P1.1 | LEWEWFEQLEEFLEWEWFEQLEEF | 3393 | 3.4 | -0.80 |
| P1.2 | EFEYEMELLWEKEFEYEMELLWEK | 3315 | 3.9 | -0.94 |
| P1.3 | EEFAMWWLMEELEEFAMWWLMEEL | 3251 | 3.4 | 0.02 |
| P1.4 | FKAKLMWWQEEKFKAKLMWWQEEK | 3271 | 9.2 | -1.14 |
| P1.5 | EESFMWKMKKEWEESFMWKMKKEW | 3341 | 6.2 | -1.52 |
| P1.6 | LKWLMFWAKEEMLKWLMFWAKEEM | 3247 | 6.3 | -0.05 |
| P1.7 | SELYWFEMMYKFSELYWFEMMYKF | 3371 | 4.5 | -0.17 |
| P1.8 | AEWSYYQLAKEYAEWSYYQLAKEY | 3124 | 4.5 | -1.05 |
| P1.9 | YEFEMLFEKKLMYEFEMLFEKKLM | 3239 | 4.8 | -0.22 |

MW; molecular weight; pI, isoelectric point; GRAVY, grand average of hydropathy value

**Figure S3. Size of liposomes and giant unilamellar vesicles (GUVs) in size dependency measurements of Figure 4.** Characterization of virus-like liposomes and giant unilamellar vesicles (GUVs). Liposomes composed of DOPC (45 mol%), sphingomyelin (SM, 25 mol%), and cholesterol (Chol, 30 mol%) were generated with diameters of 50, 100, and 200 nm by extrusion and GUVs (~5.8 µm) by the papyrus method. Shown are mean diameters ± SD measured by DLS (50–200 nm) or cell counter (GUVs).


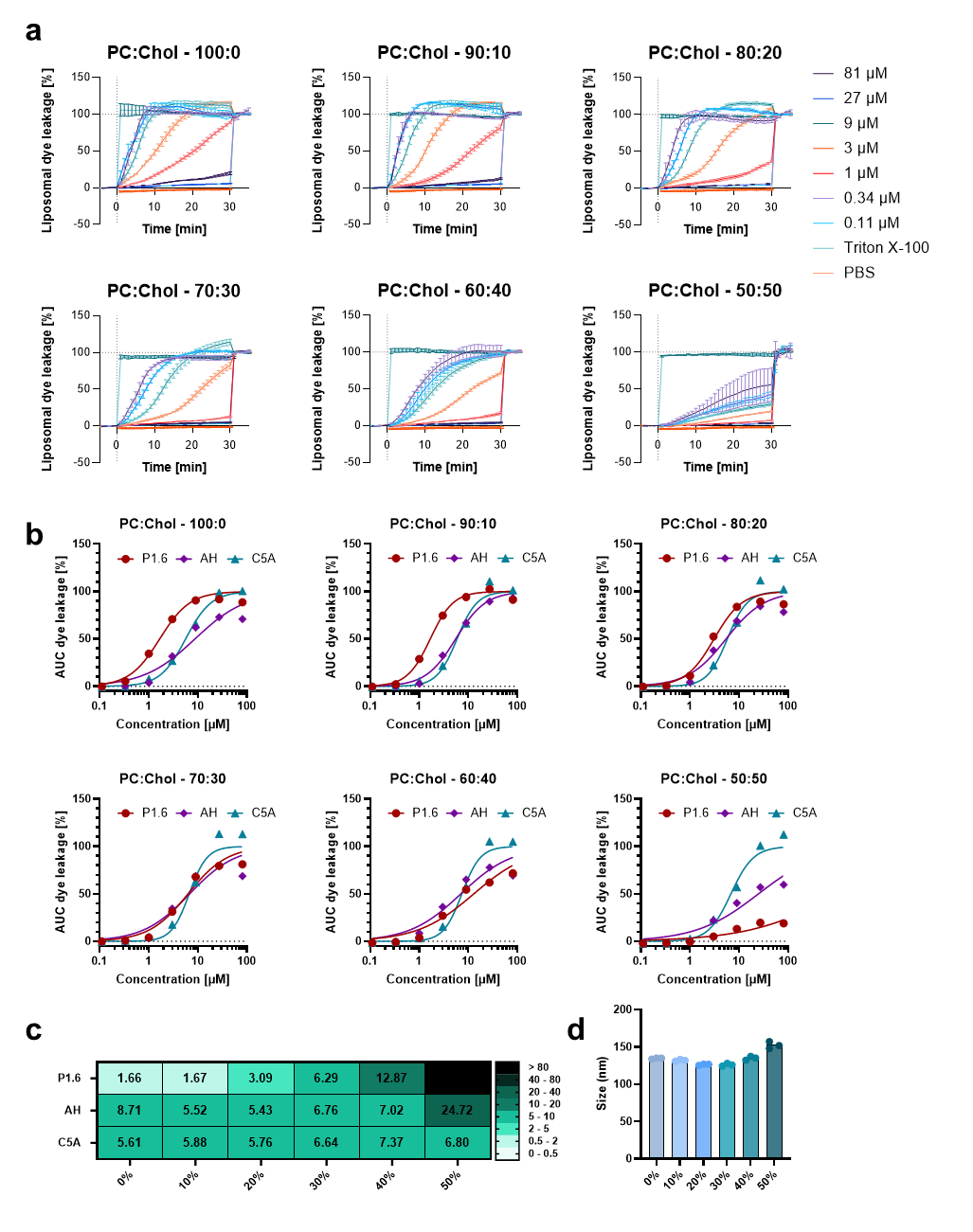


**Figure S4. Effect of increasing cholesterol concentration on the activity of P1.6. (a)** Liposomes with PC/Chol concentrations from 0-50% cholesterol were incubated with increasing peptide concentrations for 30 minutes, fluorescence was measured as described before. Time resolved leakage data. **(b)** Membrane disruption was quantified and is shown as area under the curve (AUC) after baseline subtraction derived from (a) and additional data for AH and C5A. Data represent mean ± SD of one experiment performed in triplicate. **(c)** EC₅₀ values for P1.6, AH, and C5A derived from leakage assays shown in (b). **(d)** Characterization of liposomes composed of PC and 0 - 50% cholesterol. Shown are mean diameters ± SD measured by NTA

^
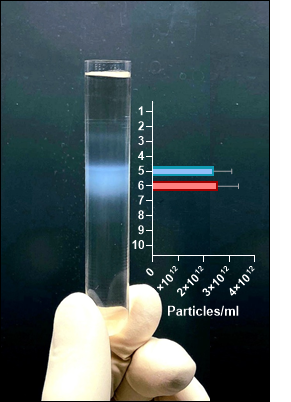
^

**Figure S5. HIV-1 purification via sucrose gradient ultracentrifugation (UZ).** HIV-1 particles were concentrated and layered on top of a discontinuous sucrose gradient containing 60% (w/v) sucrose, followed by ultracentrifugation. A distinct gray band, visible in fraction 5 and 6, indicates the concentrated viral layer. Fractions were collected sequentially from top to bottom, and particle concentrations were quantified using nanoparticle tracking analysis (NTA). The bar graph shows particle concentrations for each fraction, with fraction 5 (blue) and fraction 6 (red) containing the highest particle counts (~2.4–2.6 × 10¹² particles/mL). Fraction 5 was selected for downstream characterization by transmission electron microscopy (TEM).

**Figure S6. RAW ATR-FTIR spectrum.** 4. Colored lines represent all measured ATR-FTIR spectra that were used for the evaluation shown in Fig. 4. The DOPC 2000 µM spectrum (blue) serves as the background reference at the highest lipid concentration tested in this setup. Data represent mean ± SD from one experiment performed in triplicate.


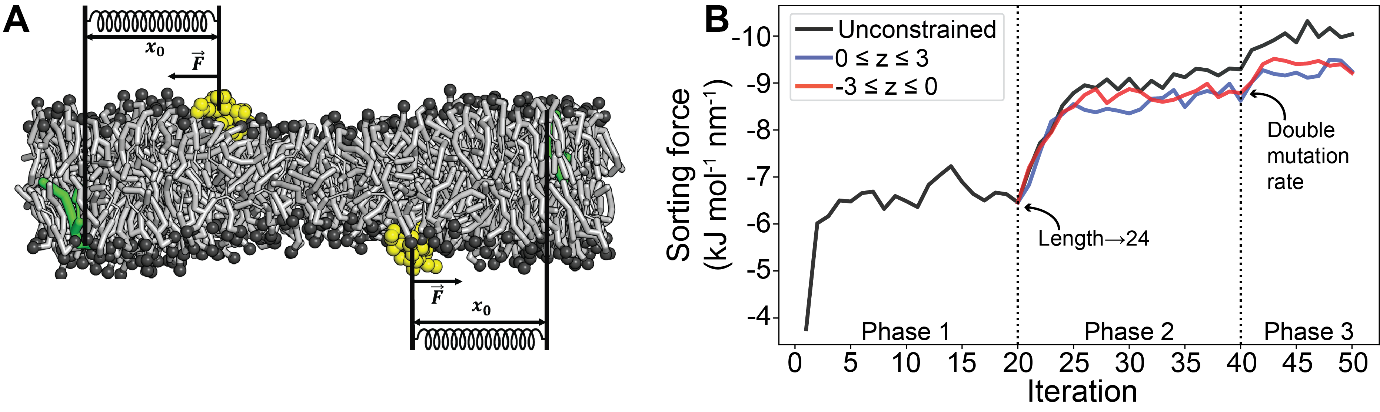


**Figure S7. Peptide fitness evaluation and evolutionary optimization in a membrane packing defect gradient. (a)** Schematic illustration of the fitness calculation used in the evolutionary design of membrane-interacting peptides. Lipid packing defects were introduced by locally thinning the DOPC bilayer, creating a gradient in membrane thickness (17). Peptides (yellow) interact with a DOPC choline bead (green) in the opposing membrane leaflet, which is restrained in the x-direction. The sorting force F = k(x − x_0_) with spring constant k=50 kJ mol−1nm−2, quantifies the deviation from the equilibrium distance x_0_, reflecting the peptide’s tendency to localize to regions of lipid packing defects. More negative values of F indicate stronger attraction toward the thinned membrane region and thus higher fitness. **(b)** Evo-MD-driven optimization of lipid packing sensing peptides. Black trace represents peptides sampled without charge (z) constraints. Blue and red traces correspond to peptides with enforced positive (0 ≤ z ≤ 3) or negative (–3 ≤ z ≤ 0) charges, respectively. The three different phases of optimization are clarified in Table S2.

**Table S2: Genetic operations**. Summary of the probabilities for the genetic operations that are applied when generating a next population of peptides

|  | Phase 1 (it 0-20) | Phase 2 (it 21-40) | Phase 3 (it 41-50) |
| --- | --- | --- | --- |
| Crossover (Pc) | 1 | 1 | 0 |
| Point mutation (Pm) | 1 | 1 | 2 |
| Deletion (Pd) | $\frac{rL_{block}-L_{min}}{L_{max}-L_{min}}$ | 0.5 | 0 |
| Insertion (Pi) | $1-\frac{rL_{block}-L_{min}}{L_{max}-L_{min}}$ | 0.5 | 0 |
| Swapping (Ps) | 0 | 0 | 1 |
| Repeat number mutation (Pr) | $\frac{1}{L_{block}}$ (+ or -) | 1 (only +) | 0 |


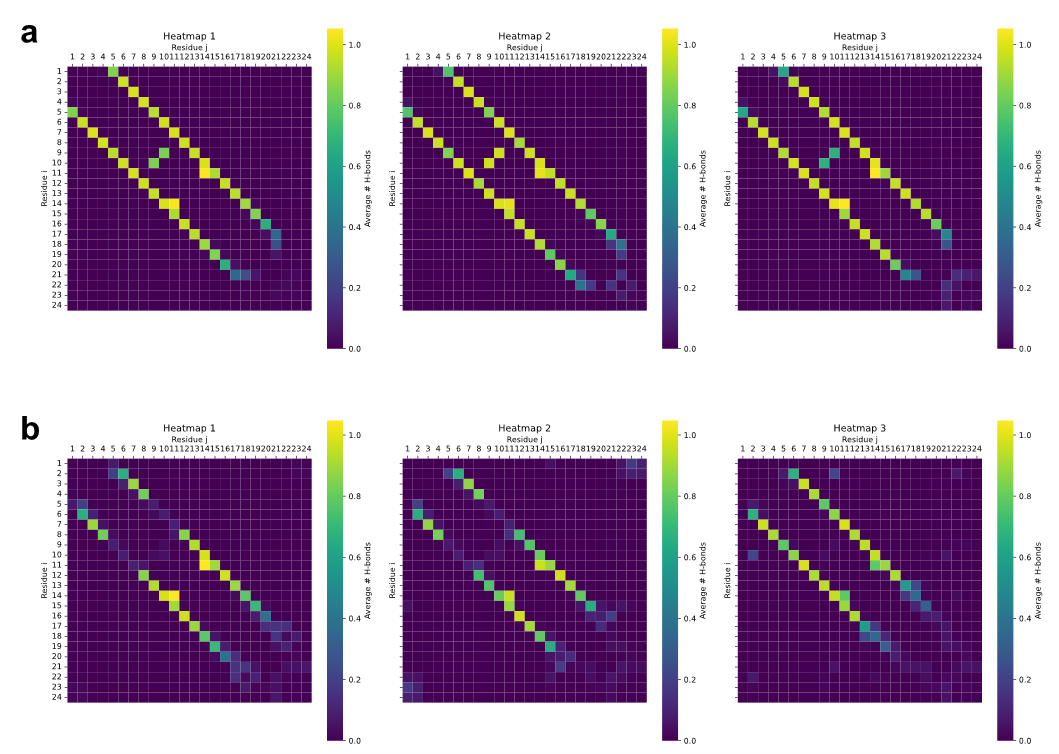


**Figure S8. Intra-peptide hydrogen bond occupancies of peptide P1.6.** The average number of hydrogen bonds between residues 'i' and 'j' are shown in the presence of a pure POPC lipid bilayer **(a)**, and in water **(b)**. The heatmap shows an i->i+4 pattern of residue-residue hydrogen bonds, characteristic for alpha-helices. For the membrane-bound system, this structure is maintained for most of the peptide except for the latter residues (20-24, "AKEEM"). In contrast, in the water system we see the presence of a kink in the alpha-helical structure around residues 7-11 as predicted by the PEP-FOLD3 server.


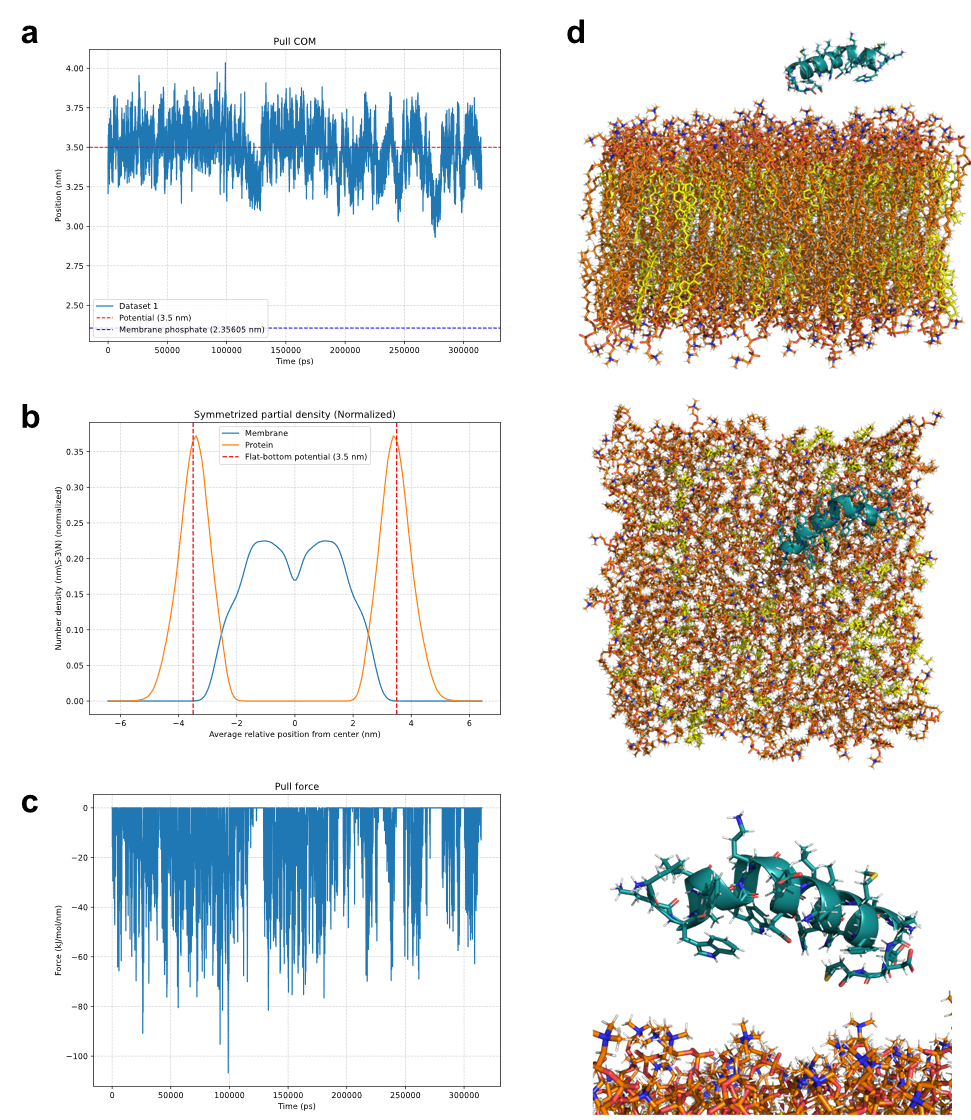


**Figure S9. Peptide P1.6 remains unbound to cholesterol-rich membranes despite an applied flat-bottom potential.** A simulation system containing POPC (70 mol%), cholesterol (30 mol%), 150 mM NaCl, and one P1.6 peptide was used to assess peptide–membrane interactions. To promote membrane association, a flat-bottom potential of 1000 kJ mol⁻¹ nm⁻² was applied between the peptide and membrane centers of mass (COMs) along the membrane normal, with zero force below a 3.5 nm COM distance. The peptide remained unbound throughout the simulation. **(a)** Peptide–membrane COM distance over time, with the flat-bottom potential (red) and average membrane phosphate plane (purple) indicated. **(b)** Symmetrized partial density profiles of peptide (yellow) and membrane (blue), showing no overlap despite the applied potential. **(c)** Force profile further confirming that P1.6 was held near, but did not interact with, the membrane. **(d)** Snapshot from a 100 ns simulation after release of the potential, showing the peptide (cyan) moving away from the DOPC:Chol (70:30 mol/mol) bilayer (orange and yellow), illustrating the unfavorable interaction with the cholesterol-rich membrane.


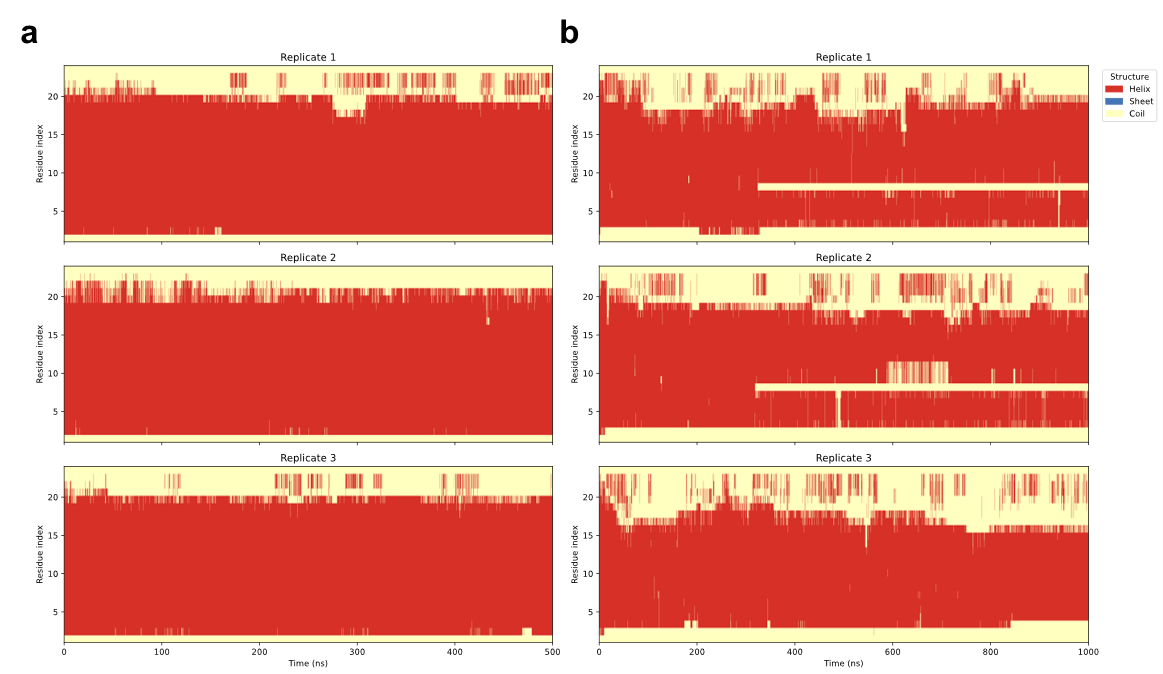


**Figure S10. Predicted secondary structures of peptide P1.6 with respect to time, as predicted by the DSSP algorithm**. Three replicates are shown per system. In the presence of a pure POPC lipid bilayer **(a)**, the peptide shows a stable helical region (red) for most of the peptide, except for the final residues (~20--24, "AKEEM"), which are predicted to be unstructured (yellow). In a solvent-only simulation **(b)** a kink forms around residues 7-11, as predicted by the PEP-FOLD3 server.
